# Exploring Stress, Fatigue, Burnout, and Resilience Among Healthcare Personnel in Southern and South-Eastern Asia: A Scoping Review

**DOI:** 10.3389/phrs.2025.1608603

**Published:** 2025-11-26

**Authors:** Kelsey G. Trulik, Vijaya A. Kumar, Wendy Wu, Muralidhar Varma, Mauli M. Patel, Kajol Manglani, Trini A. Mathew

**Affiliations:** 1 School of Medicine, Wayne State University, Detroit, MI, United States; 2 Shiffman Medical Library, Wayne State University, Detroit, MI, United States; 3 Department of Infectious Disease, Kasturba Medical College Manipal, Manipal Academy of Higher Education, Manipal, Karnataka, India; 4 Department of Internal Medicine/Pediatrics, Corewell Health West, Michigan State University, Grand Rapids, MI, United States; 5 Department of Endocrinology, MedStar Georgetown University Hospital, Washington, DC, United States

**Keywords:** healthcare personnel, healthcare workers, Southern Asia, South-eastern Asia, mental health, resilience, burnout

## Abstract

**Objectives:**

This study aims to compare methods used to measure burnout, fatigue, stress, and resilience, as well as resilience-building interventions among healthcare personnel (HCP) in Southern and South-eastern Asia. Even before COVID-19, HCP faced high levels of burnout and stress, exacerbated by the pandemic. Identifying effective resilience-building strategies is essential to supporting a healthier workforce.

**Methods:**

Studies published from January 2016 to December 2021 focusing on burnout, stress, fatigue, and resilience were included. COVIDENCE software was used for screening.

**Results:**

A total of 55 studies were included in the review. Of these 55 studies, 51 measured burnout, stress, fatigue, or resilience, using 77 different instruments. The MBI-HSS, PSS-10, BRS, Brief-COPE, and CD-RISC were the most common tools to assess burnout, stress, and individual resilience. Four studies evaluated resilience interventions, using mindfulness training, meditation, progressive muscle relaxation, and yoga.

**Conclusion:**

There are many studies assessing burnout, stress, and resilience among HCP in Southern and South-eastern Asia, yet gaps remain in identifying effective resilience-building interventions. Further research is needed to assess the impact of individual resilience on health systems resilience.

## Introduction

The World Health Organization (WHO) defines health as “a state of complete physical, mental and social wellbeing and not merely the absence of disease or infirmity” [1]. In healthcare, there is frequently an overemphasis on physical conditions, such as comorbidities, while mental health is oftentimes overlooked. The COVID-19 pandemic has highlighted the critical need for mental health and resilience in healthcare personnel (HCP).

Resilience has been a focus of research for several decades, but its importance has become particularly evident during the COVID-19 pandemic. The American Psychological Association (APA) defines resilience as “the process and outcome of successfully adapting to difficult or challenging life experiences, especially through mental, emotional, and behavioral flexibility and adjustment to external and internal demands” [2]. Smith et al. (2008) provide a more concise and unidimensional definition, describing resilience as “resistance to illness, adaptation, and thriving—the ability to bounce back or recover from stress” [3].

Stressors of HCP during COVID-19 included fear of transmission of disease to friends and family, the uncertainty of the clinical implications of the virus, and emotional exhaustion and fatigue in caring for a larger volume of sick, often critically ill, patients. Support systems and a feeling of camaraderie amongst colleagues in the field is noted as a positive coping mechanism in reducing stress, preventing burnout, and improving efficiency in the workplace [4]. Similarly, resilience can be improved by both internal factors, such as mentorship, work-life balance, spirituality, and self-reflection, as well as external systemic supports like job sharing, and structured training programs [5].

While healthcare professions can be deeply rewarding, they are also a significant source of stress, often leading to burnout. The APA defines burnout as a state of “physical, emotional, or mental exhaustion accompanied by decreased motivation, lowered performance, and negative attitudes toward oneself and others” [6]. Stress typically arises after prolonged periods of intense physical or mental exertion, especially when faced with overwhelming workloads or pressure.

Even before the COVID-19 pandemic, healthcare workers faced elevated risks of emotional exhaustion, fatigue, stress, burnout, depression, anxiety, substance abuse, and suicide [7]. The pandemic further exacerbated these issues, with increased workloads, a global sense of hopelessness, and social isolation leading to worsened mental health outcomes [8]. Frontline workers were especially susceptible to the pandemic’s negative effects, with numerous studies indicating higher rates of anxiety, emotional exhaustion, depression, mental distress, and fatigue [9]. This strain resulted in decreased job satisfaction, poorer patient outcomes, significant challenges in staging healthcare settings, and financial burdens.

Many scales have been developed for measuring perceived stress and burnout, these include the perceived stress scale (PSS-10) and Maslach Burnout Inventory Human Services Survey (MBI-HSS) [10, 11]. The PSS-10 and MBI-HSS are widely used, reliable, and valid instruments for assessing stress and burnout, respectively [12, 13].

In 2008, the Brief Resilience Scale (BRS) was designed to assess an individual’s capacity to recover from health-related stressors [3]. Before the development of the BRS, several other assessments, such as the Connor-Davidson Resilience Scale (CD-RISC) [14], Brief Coping Orientation to Problems Experienced (Brief-COPE) [15], and Hospital Anxiety and Depression Scale (HADS) [16] were commonly used. These instruments offered in-depth assessments of personal traits that may promote positive adaptation. However, they did not directly assess resilience itself, as previously defined [3]. The BRS is a reliable and valid instrument whose widespread application has revealed poor resilience in HCPs that negatively impacts wellbeing, leading to increased depression, anxiety, fatigue, burnout, and stress. On the other hand, more resilience has been found to positively correlate with positive wellbeing indicators, such as life satisfaction, and good coping [17].

During the COVID-19 pandemic, the core authors in the USA and India recognized the urgent need for a more profound assessment of resilience and explored potential interventions to strengthen resilience among healthcare staff. This scoping review focuses on individual resilience, recognizing that while health systems resilience is critical, it is the resilience of individuals within those systems that often determines the success of the response to a crisis.

The primary aim is to identify and compare methods used to measure the level of stress, fatigue, and burnout, as well as methods used to measure resilience in individuals. The secondary aim is to identify and compare interventions used to build resilience among HCP in Southern and South-eastern Asian countries.

### Inclusion Eligibility Criteria

#### Participants

We included all HCP as defined by the Centers for Disease Control and Prevention (CDC) [18]. We excluded medical students from our inclusion participants.

#### Concept

This scoping review focused on studies examining individual resilience, defined by the National Academy of Sciences as “the ability to prepare and plan for, absorb, recover from, or more successfully adapt to actual or potential adverse events” [19]. We included studies that explored factors contributing to or influencing resilience at the individual level. We excluded studies that focused on the resilience of health systems or organizational structures.

#### Context

We included all South-eastern Asian and Southern Asian countries as defined by the United Nations [20] and World Health Organization [21]. Setting of studies include Academic Centers (University, College), Non-Academic Hospitals, and Community. All studies included were published after January 2016. Pre-COVID-19 studies collected data before March 11, 2020, the date the WHO declared COVID-19 a global pandemic [22]. Intra-COVID-19 studies collected data on or after this date.

#### Types of Sources

This scoping review considered both experimental and quasi-experimental study designs including randomized controlled trials, non-randomized experimental trials, before and after studies and interrupted time-series studies. In addition, analytical observational studies including retrospective cohort studies, case-control studies, and analytical cross-sectional studies were considered for inclusion. This review also considered descriptive observational study designs including case series, individual case reports and descriptive cross-sectional studies for inclusion. Qualitative studies were considered. In addition, systematic reviews that meet the inclusion criteria were considered, depending on the research question. Text and opinion papers were considered for inclusion in this scoping review. Clinical trials were excluded, in addition to editorials, theses or dissertations, commentaries, conference proceedings, and book chapters. Studies not published in journals, studies that were withdrawn, and studies where the full text was not available were excluded.

## Methods

### Search Sources and Strategy

A comprehensive and systematic literature search was conducted to identify peer-reviewed studies through seven databases (PubMed, PsycInfo, EMBASE, Scopus, Web of Science, CINAHL, Cochrane Library) from 2016 to Dec 22, 2021. The search terms reflected three concepts 1) resilience, 2) healthcare personnel, and 3) Southern and South-eastern Asian countries. Terminology for healthcare personnel was derived from PubMed Medical Subject Headings (MeSH), EMBASE Emtree, and CINAHL Subject Headings. The preliminary search terms were developed and refined through consultation with a core team member. The multiple combinations of search terms to the concepts were executed using Boolean operators (see the complete PubMed search in Supplementary Appendix as an example). The search results were restricted to journal articles published in English, excluding editorials, comments, letters, news, conference proceedings, book chapters, and dissertations using database built-in filters. The search strategies in PubMed were adapted to other databases mentioned above. To locate additional articles not captured by bibliographic database searching, a gray literature search was conducted, and a few hand-picked relevant articles were also added to the search results for screening. A protocol does not exist for this scoping review. This review was performed following PRISMA-ScR guidelines [23].

### Screening

Search results were imported into COVIDENCE systematic review software (Veritas Health Innovation, Melbourne, Australia) and underwent two rounds of screening based on their (1) title and abstract and (2) full text. After deduplication, the three members (KGT, KM, and MMP) conducted screening and excluded studies based on exclusion criteria (e.g., no full text available, resilience of health systems, wrong settings or study designs, wrong population, etc.). The discrepancies were resolved through discussion and consensus by TAM, VAK, and MV.

### Data Extraction

**FIGURE 1:**
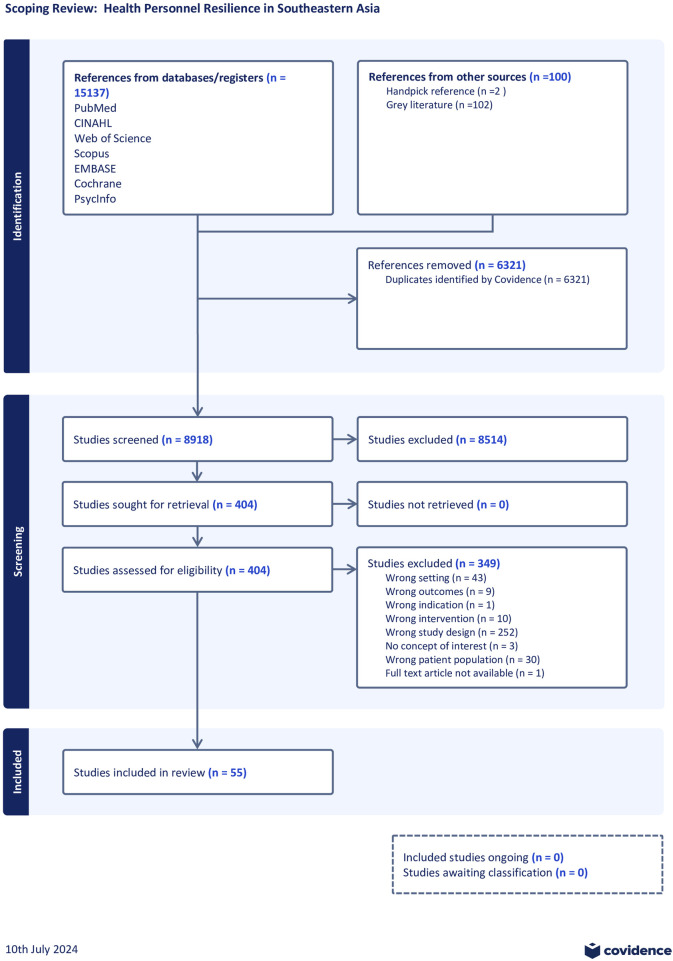
PRISMA flowchart illustrating search strategy and selection (Southern and South-eastern Asia, 2016–2021).

Data extraction was performed independently using Excel (by KGT). Data extracted includes specific details about the study settings, context, aims, time frame, participants, study design, study methods, outcomes, and key findings relevant to the review questions. A PRISMA flow diagram is listed below (Figure 1).

## Results

**FIGURE 2:**
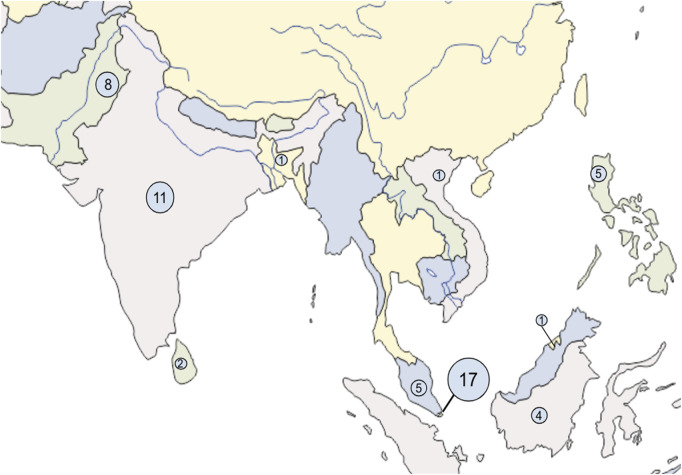
Geographic setting of included studies (Southern and South-eastern Asia, 2007–2025).

A total of 8918 studies were screened, and 55 studies were included in this study based on inclusion and exclusion criteria. A total of 10 countries were represented, with the greatest number of studies from Singapore (Figure 2). Tables 1A, B shows a summary of the 51 studies with a total of 77 different instruments used. The MBI-HSS (10 studies) and PSS-10 (5 studies) were the most common tools to assess burnout and stress, respectively. The BRS (8 studies), Brief-COPE (7 studies), and CD-RISC (7 studies) were the most common tools to measure resilience. Table 2 shows a summary of the four studies that evaluated resilience interventions.

**TABLE 1A:** Summary of methods studies reviewed using the most common tools to assess burnout and stress: MBI-HSS and PSS-10 (Southern and South-eastern Asia, 2016–2021).

Study ID	Title	Country and Setting of Study	Aim of study	Study design	Method used to determine level of burnout, stress, or fatigue	Population description	Outcome
Yang 2017 [25]	Is Mindfulness Associated with Stress and Burnout Among Mental Health Professionals in Singapore?	Singapore; Community	To investigate whether mindfulness is associated with stress and burnout among healthcare professionals working in a mental health setting in Singapore.	Cross sectional study	OLBI; PSS-10	Physicians; Nurses; Therapists; Other (Social worker, case manager, psychologist) n = 224 71.7% Female <25 years - 7.8% 25-30 years - 29.7% 30–35 years - 21.9% >35 years - 40.6%	This study showed that mental health professionals in Singapore who have higher levels of mindfulness also have lower levels stress and burnout (disengagement and exhaustion). Future longitudinal research is warranted to better understand the directionality of these associations, with implications for the development of interventions aimed to reduce stress and burnout within this population.
Zafar 2016 [26]	Workplace Violence and Self-Reported Psychological Health: Coping with Post-Traumatic Stress, Mental Distress, and Burnout Among Physicians Working in the Emergency Departments Compared to Other Specialties in Pakistan	Pakistan; Non-academic hospitals	To measure the prevalence of workplace violence (WPV) among emergency physicians in 4 of the largest hospitals in Karachi, Pakistan. To measure the association between the experience of WPV and self-report of post-traumatic stress disorder (PTSD), depression, anxiety, and burnout. To compare the same factors across medical specialties. To explore the coping strategies used by physicians in dealing with job-related stressor.	Cross sectional study	Physicians’ experience of WPV were measured using a survey instrument developed by the Joint Programme on Workplace Violence in the Health Sector of the International Labor Office, the International Council of Nurses, the World Health Organization, and the Public Services International. The questionnaire has three sections: 1) questions regarding personnel and workplace characteristics; 2) questions about physical violence in the workplace, and 3) questions related to verbal abuse. The primary question of interest was whether, in the last 12 months, the respondent had been physically attacked or verbally abused in the workplace. PTSD symptoms were screened using PCL-C. Burnout was measured using MBI-HSS. Symptoms of mental distress, anxiety, and depression were screened using the GHQ-12.	Physicians n = 179 58.7% Female <30 years - 65.4% ≥30 years - 34.6%	Experience of WPV was not uniform across specialties but was generally high among Pakistani physicians. Prevention of WPV should be a high priority for health care policy makers.
Lal 2020 [35]	The Prevalence, Determinants and the Role of Empathy and Religious or Spiritual Beliefs on Job Stress, Job Satisfaction, Coping, Burnout, and Mental Health in Medical and Surgical Faculty of a Teaching Hospital: A Cross-Sectional Survey	India; Academic (University, College)	To survey the perspectives of HCWs on what has impacted their morale and to assess their anxiety levels before and after screening duties in NCID	Cross sectional study	CJSSQ; GHQ-12; JSPE; MBI-HSS	Physicians; Other (Medical Professors and Tutors) n = 345 38.8% Female Mean age = 40.5 years	This study provides data that will inform the design and implementation of interventions to reduce job stress and burnout and improve retention of faculty.
Mahmood 2021 [36]	Triggering and Protective Factors of Burnout in Medical Resident Physicians in a Lower-Middle-Income Country: A Cross-Sectional Study	Pakistan; Academic (University, College)	To determine the burden of burnout among internal medicine residents and to identify triggering and protective factors associated with burnout	Cross sectional study	An abbreviated version of the MBI-HSS was used to measure burnout. The survey also included the triggering and protective factors which were scored on a five-item Likert scale.	Residents n = 71 71.8% Female Mean age = 28.3 years (SD 3.1)	More than a third of medicine residents suffered from burnout. We need to focus on rejuvenating activities for medicine residents to decrease burnout among them. If not addressed adequately this may result in a compromise in the quality of care being provided to patients.
Chong 2021 [41]	Burnout and Resilience Among Pharmacy Technicians: A Singapore Study	Singapore; Community	To quantify burnout in pharmacy technicians in patient care sectors in Singapore and to explore factors that may be associated with burnout	Cross sectional study	MBI-HSS; A checklist with common factors was provided and free text responses were allowed to elicit possible reasons for burnout and coping strategies according to the respondents’ self-perceptions. burnout	Pharmacists n = 725 85.5% Female Mean age = 34 years (SD 12)	Burnout affects most PTs in Singapore and is primarily driven by workload and nature of their work, low resilience, and poor social support structures. National and organizational efforts are needed to arrest the vicious cycle that propagates burnout in PTs.
Chui 2021 [48]	The COVID-19 Global Pandemic and its Impact on the Mental Health of Nurses in Malaysia	Malaysia; Community	To assess the perceived stress, stress symptoms, and levels of depression experienced by nurses, their coping strategies, and the association of stress and depression with their demographic profiles given Malaysia’s unique ethnic pluralism	Cross sectional study	The questionnaire consisted of four main parts. Part I comprised questions that solicited the demographic, social, and job-related characteristics of the nurses. Parts II-III were PSS-10 and MDI.	Nurses n = 859 Mean age = 32.7 years (SD 6.9)	The findings of the study provide insight into the mental health and coping strategies of nurses actively involved in caring for patients with suspected or confirmed COVID-19 in Malaysia. This would be of tremendous help to nursing administrators in implementing mental health services for nurses during and following the COVID-19 global pandemic.
Gupta 2020 [50]	Pandemic and Mental Health of the Front-Line Healthcare Workers: a Review and Implications in the Indian Context Amidst COVID-19	Canada, Taiwan, China, South Korea, Saudi Arabia, Sierra Leone, India; Academic (University, College)	To review the available literature on mental health aspects of the pandemic on the front-line HCWs, discuss some of the contentious issues, and provide future directions particularly concerning COVID-19 in the Indian context and apply to other developing nations with low- resource healthcare facilities.	Systematic review	ABQ; AS; BAI; BDI; DASS-21; DTS; GAD-7; GHQ-12; IES-R; ISI; MERS-CoV staff questionnaire; MBI-HSS; PHQ-9; PSQI; PSS-10; SCL-90-R; SS; STAI; STAXI; Self-rated attitude scale towards SARS; Questionnaire for SARS-related risk perception; SARS-related stress reactions questionnaire	Physicians; Nurses; Nursing Assistants; Residents; Pharmacists	Current review attempts to highlight the mental health aspects of the pandemic on the front- line HCWs, discusses some of the contentious issues and provides future directions particularly concerning COVID-19 in the Indian context and other low- resource countries.
Chew 2020 [51]	Perceived Stress, Stigma, Traumatic Stress Levels and Coping Responses amongst Residents in Training across Multiple Specialties during COVID-19 Pandemic—A Longitudinal Study	Singapore; Academic (University, College)	To examine how the earlier psychological responses within residents in training across multiple specialties have evolved over the course of the pandemic, as well as the relevant associated demographic factors and coping strategies.	Longitudinal study	HWSS; IES-R; PSS-10	Physicians n = 221 49.8% Female Mean age = 30.8 years (SD 2.9)	Residency programs should encourage active coping strategies (e.g., seeking social support, positive thinking, problem solving) among residents, and proactively identify residents who may be at higher risk of psychological sequelae due to circumstances that contribute to isolation.
Jose 2020 [53]	Burnout and Resilience among Frontline Nurses during COVID-19 Pandemic: A Cross-sectional Study in the Emergency Department of a Tertiary Care Center, North India	India; Academic (University, College)	To determine burnout and resilience and its associated factors among the frontline nurses who provide direct care for the patients in the emergency department of a tertiary care center in North India.	Cross sectional study	MBI-HSS	Nurses n = 120 73.3% Female Mean age = 29 years (SD 4.4)	Effective interventions for improving resilience are needed to relieve nurses’ burnout and workplace stressors. Also, the administration should ensure a healthy workplace and adopt a positive attitude and harmonious relationship with the frontline workers in the mitigation of the pandemic.
Ma 2021 [58]	Curbing Nurses' Burnout During COVID-19: The Roles of Servant Leadership and Psychological Safety	Pakistan; Community	To examine the role of servant leadership through the mechanism of psychological safety in curbing nurses' burnout during the COVID-19 pandemic.	Cross sectional study	Researchers designed three separate survey forms to capture independent, mediator, and dependent variables on Google docs. At the first time point demographic data and a rating of the servant leadership of their supervisors/head nurses was obtained. After 10 days, at the second time point, questions about psychological safety were asked from the same nurses who participated in the first survey. A 7-point Likert scale (1 = strongly disagree, 7 = strongly agree) was employed to solicit the participants’ responses. The nurses’ servant leadership was evaluated by the nurses on a seven-item measure of the global servant leadership (Liden et al., 2015). Psychological safety was evaluated with a three-item scale adapted by Detert and Burris (2007) from the original measure of psychological safety by Edmondson (1999). Burnout was assessed by MBI-GS.	Nurses n = 443 94.1% Female 18-30 years - 42% 31–45 years - 39% 45+ years - 19%	Servant leadership significantly reduces nurses’ burnout, and psychological safety mediates this relationship.
Ong 2021 [59]	An Evaluation of the Performance of Five Burnout Screening Tools: A Multicentre Study in Anaesthesiology, Intensive Care, and Ancillary Staff	Singapore; Non-academic hospitals	To compare the diagnostic performance of five burnout screening tools, including a novel rapid burnout screening tool (RBST).	Cross sectional study	The 36-item electronic survey consisted of three parts. The first part comprised the MBI-HSS. The second part contained the RBST and a single item burnout question (SIBOQ).	Physicians; Nurses; Nursing Assistants; Residents; Support Staff n = 493 77.7% Female 20–29 years - 27.8% 30-39 years - 49.1% 40–49 - 15.6% 50–59 - 4.9% ≥60 years - 2.6%	Brief screening tools detect burnout albeit with a wide range of accuracy. This can strain support services and resources. The RBST is a free screening tool that can detect burnout with a high degree of accuracy
Sugiharto 2020 [62]	Coping Strategies Potentially Reduce Burnout Syndrome in Anesthesiologists.	Indonesia; Academic (University, College)	To determine the relation between coping and burnout in anesthesiologists.	Cross sectional study	MBI-HSS	Residents n = 60 20% Female Mean age = 32.2 years (SD 3.08)	Coping is correlated with burnout in anesthesiologists. Approach coping reduces the incidence of burnout in anesthesiologists. Working hours as a burnout aggravating factor is also significantly correlated with burnout.
Ho 2021 [72]	Levels of Burnout and its Association with Resilience and Coping Mechanisms Among Orthopedic Surgery Residents: a Single Institution Experience from Singapore.	Singapore; Academic (University, College)	To describe the levels of burnout and psychological morbidity among orthopedic surgery residents in an ACGME-I accredited program at a single institution. The secondary aim was to determine the associations between levels of burnout and resident characteristics, resilience, and coping mechanisms.	Cross sectional study	GHQ-12; MBI-HSS; 25-questions regarding subjective stressors, which included personal and work-life conflicts, work environment, surgical complications as well as financial and employment prospects.	Residents n = 44 9.1% Female Mean age = 31.4 years (SD 2.3)	Burnout was high in our ACGME-I accredited program. Stressors associated with higher burnout included feeling of inadequate sleep, poor work-life balance, poor relationships with fellow residents/faculty and financial pressures. Residents should be educated on protective coping mechanisms and regular screening to detect burnout should be performed.
Riaz 2021 [73]	Stress and Coping Among Surgery Residents in a Developing Country	Pakistan; Academic (University, College)	To measure the stress level among the surgical residents, identify factors within the learning and work environment that cause stress, and identify different strategies that the residents use habitually to cope with these stresses. The study also explored the relationship between stress levels and continuous assessment scores during residency training.	Cross sectional study	PSS-10	Residents n = 68 16.2% Female	Residents in surgical residency programs at AKU perceived themselves to be under stress. Periodic evaluation can help identify residents with high stress levels, and thus specific strategies can be adopted. Academia and hospital administration should join hands in developing interventions to enable residents cope with the situation.

**TABLE 1B:** Summary of methods studies reviewed using the most common tools to measure resilience: BRS, Brief-COPE, and CD-RISC (Southern and South-eastern Asia, 2016–2021).

Study ID	Title	Country and Setting of Study	Aim of study	Study design	Method used to measure resilience	Population description	Outcome
Ang 2020 [32]	Differing Pathways to Resiliency: A Grounded Theory Study of Enactment of Resilience Among Acute Care Nurses	Singapore; Community	To generate a comprehensive account of the experiences of nurses as they cope with stress and demands of work, and to develop knowledge of the phenomenon of resilience among nurses	Qualitative research	Participants were recruited based on their scores on the CD-RISC-10. Interview questions with nurses asked What do you do to cope with stressful situations? How would you guide someone more junior to cope with work stress? What does the word “resilience” mean to you? Do you think you are resilient? Why? Do you think resilience can be developed? The initial interviews were free-flowing, and as the interviews went on, probing questions were used to obtain more comprehensive and in-depth information.	Nurses n = 18 83.3% Female Mean age = 38 years	Highly resilient nurses tend to adopt active coping mechanisms, whereas nurses who have low resilience tend to undertake passive measures to let nature runs its course. The emerging theory provided an understanding of the different pathways to resiliency and how nurse leaders can potentially develop and grow the level of resiliency among nurses.
Chang 2019 [33]	Resilience and Associative Stigma Among Mental Health Professionals in a Tertiary Psychiatric Hospital: a Cross-Sectional Study in Singapore	Singapore; Non-academic hospitals	To examine correlates of resilience and its association with associative stigma among mental health professionals working at the Institute of Mental Health (IMH), which is the only tertiary psychiatric hospital in Singapore.	Cross sectional study	BRS (mean 3.59, SD 0.64)	Physicians; Nurses; Other (Psychologists, Pharmacists, Occupational Therapists, Physiotherapists, Case Managers, Medical Social Workers) n = 462 63% Female Mean age = 36.4 years (SD 10.6)	The present finding suggests that resilience building programs among mental health workers should target those of the younger age group, and that addressing the issue of associative stigma is essential.
Ying 2020 [37]	Nursing Practice Environment, Resilience, and Intention to Leave Among Critical Care Nurses	Malaysia; Academic (University, College)	To assess the association between perceived nursing practice environment, resilience, and intention to leave among critical care nurses and to determine the effect of resilience on intention to leave after controlling for other independent variables.	Cross sectional study	CD-RISC-25 (mean 68.24, SD 11.33, range 43–96); PES-NWI-12; The future job plan survey was used to assess ITL as it measures nurses' future job plan for the next 3 years based on eight items using a 5-point Likert scale (1 = “strongly disagree” to 5 = “strongly agree”).	Nurses n = 229 93.9% Female ≥25 years - 36.7% 26–30 years - 41.9% >30 years - 21.4%	This study found that an unfavorable practice environment is a strong predictor of intention to leave; however, further exploration is needed to explain the higher likelihood of expressing intention to leave among CCNs when their resilience level increases.
Ang 2018 [39]	Association Between Demographics and Resilience – a Cross-Sectional Study Among Nurses in Singapore	Singapore; Academic (University, College)	To provide an overview of the level of resilience among nurses in Singapore and examine associations between various demographic variables and resilience level.	Cross sectional study	CD-RISC-10 (mean 25.9, SD 6.0, range 4-40)	Nurses n = 1338 93% Female ≥27 years - 29% 27.1-40 years - 46% 40.1+ years - 24%	The experience of life events (as exemplified by marital status, age and working experience) was associated with higher resilience levels.
Chong 2021 [41]	Burnout and Resilience Among Pharmacy Technicians: A Singapore Study	Singapore; Community	To quantify burnout in pharmacy technicians in patient care sectors in Singapore and to explore factors that may be associated with burnout	Cross sectional study	BRS (mean 3.2, SD 0.6)	Pharmacists n = 725 85.5% Female Mean age = 34 years (SD 12)	Burnout affects most PTs in Singapore and is primarily driven by workload and nature of their work, low resilience, and poor social support structures. National and organizational efforts are needed to arrest the vicious cycle that propagates burnout in PTs.
Khan 2021 [44]	Psychological Distress Among Bangladeshi Physicians: Roles of Perceived Stigma, Fear of Infection and Resilience in the Context of Covid-19 Pandemic	Bangladesh; Community	The investigate the association among perceived stigma, fear of infection, resilience, and psychological distress among physicians during the Covid-19 pandemic	Cross sectional study	BRS (mean 3.22, SD 0.438)	Physicians n = 209 48.8% Female 31–40 years - 49.3% 21–30 years - 47.4%	The findings highlighted the importance of psychological care for the physicians and designing effective awareness programs for the community people to intervene in the stigmatization circle.
Rishipathak 2021 [45]	Psychological Resilience Towards COVID-19 Amongst Emergency Medical Professionals in Pune (India)	India; Academic (University, College)	To assess the baseline psychological resilience amongst Emergency Medical Professionals actively working in the care of COVID-19 patients for more than a year.	Descriptive Study	BRS includes questions regarding accepting and coping with the COVID situation. AITCS assesses work pride and meaning, feeling valued and supported. MOS-SS and the GQ emphasized on positive emotions, perceived social support, protective psychosocial characteristics.	Physicians n = 120 68% Female 21–25 years - 59% 26–30 years - 34% >30 years - 7%	The findings indicate that even after a year of serving COVID 19 patients, Emergency Medical Professionals demonstrate a high degree of resilience. Yet there are areas requiring improvement which need to be focused upon immediately in the interest of the mental well-being of Emergency Medical Professionals.
Seng 2021 [46]	Resilience and Stress in Frontline Social Workers During the COVID-19 Pandemic in Singapore.	Singapore; Non-academic hospitals	To examine the psychological distress of frontline social workers and their resilience during the pandemic. To explore the association between psychological distress and resilience as well as the role of organizational support and other demographic variables that mitigate psychological distress.	Qualitative research	CD-RISC-25 (mean 68.15, SD 12.43)	Other (Social workers) n = 308 74% Female 21–29 years - 51.6% 30–39 years - 30.5% 40–49 years - 12.3% 50–59 years - 4.2% 60+ years - 1.3%	Organizations must be mindful that support can help frontline staff who are usually younger and less experienced during challenging times such as the pandemic. Building the resilience of social workers will prepare them for their daily challenges and those that accompany unexpected situations.
Sinha 2021 [47]	A Cross-Sectional Online Survey of the Relationship Between the Psychological Impacts of Coronavirus Disease 2019 (COVID–19) Lockdown and the Resilience Among Physiotherapy Professionals in India	India; Community	To assess the relationship between psychological impact of COVID-19 lockdown in the form of depression and anxiety, and the resilience among physiotherapists across India.	Cross sectional study	BRS (mean 3.2, SD 0.43)	Therapists n = 378 53.4% Female 21–35 years - 60.6% 36–45 years - 31.5% 46–60 years - 7.9%	Programs to strengthen resilience should be priority. In longer run, increasing resilience of physiotherapists can have mental health promoting value during the stressful event of COVID-19 lockdown.
Chui 2021 [48]	The COVID-19 Global Pandemic and its Impact on the Mental Health of Nurses in Malaysia	Malaysia; Community	To assess the perceived stress, stress symptoms, and levels of depression experienced by nurses, their coping strategies, and the association of stress and depression with their demographic profiles given Malaysia’s unique ethnic pluralism	Cross sectional study	Brief-COPE	Nurses n = 859 Mean age = 32.7 years (SD 6.9)	The findings of the study provide insight into the mental health and coping strategies of nurses actively involved in caring for patients with suspected or confirmed COVID-19 in Malaysia. This would be of tremendous help to nursing administrators in implementing mental health services for nurses during and following the COVID-19 global pandemic.
Chew 2020 [51]	Perceived Stress, Stigma, Traumatic Stress Levels and Coping Responses amongst Residents in Training across Multiple Specialties during COVID-19 Pandemic—A Longitudinal Study	Singapore; Academic (University, College)	To examine how the earlier psychological responses within residents in training across multiple specialties have evolved over the course of the pandemic, as well as the relevant associated demographic factors and coping strategies.	Longitudinal study	Brief-COPE	Physicians n = 221 49.8% Female Mean age = 30.8 years (SD 2.9)	Residency programs should encourage active coping strategies (e.g., seeking social support, positive thinking, problem solving) among residents, and proactively identify residents who may be at higher risk of psychological sequelae due to circumstances that contribute to isolation.
Jose 2020 [53]	Burnout and Resilience among Frontline Nurses during COVID-19 Pandemic: A Cross-sectional Study in the Emergency Department of a Tertiary Care Center, North India	India; Academic (University, College)	To determine burnout and resilience and its associated factors among the frontline nurses who provide direct care for the patients in the emergency department of a tertiary care center in North India.	Cross sectional study	CD-RISC-25 (mean 77.77, SD 12.41)	Nurses n = 120 73.3% Female Mean age = 29 years (SD 4.4)	Effective interventions for improving resilience are needed to relieve nurses’ burnout and workplace stressors. Also, the administration should ensure a healthy workplace and adopt a positive attitude and harmonious relationship with the frontline workers in the mitigation of the pandemic.
Khalid 2021 [54]	Psychological Resilience, Burnout, and Secondary Traumatic Stress among Doctors in Covid-19 Pandemic.	Pakistan; Academic (University, College)	To study the relationship between psychological resilience, burnout, and secondary traumatic stress among doctors during the COVID-19 pandemic. It also identified the mediating effect of burnout between the relationship of psychological resilience and secondary traumatic stress.	Other: Descriptive study	BRS (mean 3.81, SD 0.95)	Physicians n = 100 62% Female Age range 25-40 years	It is concluded that psychological resilience has a significant negative relationship with burnout and secondary traumatic stress. Future research can design emotional coping strategies and should try to promote programs that can help doctors to enhance resilience so it helps them combat their stress and burnout.
Kumar 2021 [55]	A Cross-Sectional Study on Mental Health and Cardiovascular Reactivity Among Fresh Resident Doctors During COVID-19 Pandemic in India	India; Academic (University, College)	To assess the mental health and cardiovascular reactivity among the resident doctors working in the capital city of India, Delhi	Cross sectional study	CD-RISC-25 (COVID ward doctors mean 64.84, SD 6.33; non-COVID ward doctors mean 72.78, SD 5.57); SSS	Residents n = 162 46.4% Female Age range 25-30 years	The findings suggested high levels of training, resilience helpful social support and unbiased work culture were necessary to health care workers engaged in public health emergence.
Saleem 2020 [60]	Self-control Mediates the Relationship between Psychosocial Strengths and Perceived Severity of COVID-19 among Frontline Healthcare Professionals of Pakistan: A Single Center Experience	Pakistan; Academic (University, College)	To examine the relationship between psychosocial strengths (resilience, self-efficacy beliefs and social support) and perceived severity of COVID-19 and also to gauge the mediating role of self-control among frontline health care professionals of Pakistan.	Cross sectional study	BRS; BSCS; BSSC	Physicians; Residents n = 284 38% Female	In the time of pandemic, medical professionals are working as frontline force and can have several uncertainties regarding the risk associated with outbreak of COVID-19. This study concludes psychosocial strengths can play a significant role in subsiding the risk associated with severity of disease. Whereas self-control can significantly contribute to buffer the negative influence of COVID-19 among frontline medical professionals. In line with findings of this study, there is a dire need to initiate psychotherapeutic studies for medical professionals to boost up their psychosocial strengths that would make them resilient against COVID-19.
Setiawati 2021 [61]	Anxiety and Resilience of Healthcare Workers During COVID-19 Pandemic in Indonesia	Indonesia; Non-academic hospitals	To determine the level of resilience and anxiety in healthcare workers who represent important aspects in handling COVID-19 outbreaks and to find out whether there is a correlation between the level of resilience and anxiety in healthcare workers in Indonesia during the COVID-19 pandemic.	Cross sectional study	CD-RISC-25 (mean 69, SD 15.823)	Nurses; Pharmacists; Medical Technicians; Support Staff; Other (Midwives, Physiotherapists, Nutritionists, Medical Records Officers, Psychologists, Laboratory Staff) n = 227 83.3% Female Mean age = 39.67 years (SD 9.43)	A significant correlation was found between the level of resilience and anxiety experienced by healthcare workers during the COVID-19 pandemic. The lower the resilience, the higher the anxiety experienced.
Sugiharto 2020 [62]	Coping Strategies Potentially Reduce Burnout Syndrome in Anesthesiologists.	Indonesia; Academic (University, College)	To determine the relation between coping and burnout in anesthesiologists.	Cross sectional study	Brief-COPE	Residents n = 60 20% Female Mean age = 32.2 years (SD 3.08)	Coping is correlated with burnout in anesthesiologists. Approach coping reduces the incidence of burnout in anesthesiologists. Working hours as a burnout aggravating factor is also significantly correlated with burnout.
Xuan 2021 [64]	Modifiable Factors Influencing Resilience among Medical Interns	Malaysia; Non-academic hospitals	To find out the resilience level of medical interns in Malaysia and its associated factors.	Cross sectional study	Brief-COPE; CD-RISC-10 (mean 28.6, SD 6.33, range 9-40); DUREL; PHPQ; USMEQ-I	Physicians n = 524 66.6% Female Median age = 26 years	Factors affecting resilience among medical interns include modifiable factors such as coping styles and involvement in sports. The findings could guide targeted intervention to promote during medical schools or internship preparation programs to increase resilience among medical interns.
Labrague 2021 [9]	Pandemic Fatigue and Clinical Nurses’ Mental Health, Sleep Quality and Job Contentment During the Covid-19 Pandemic: The Mediating Role of Resilience	Philippines; Community	To assess the extent to which resilience influences nursing outcomes during a pandemic and will help to formulate interventions that better support the mental health and overall well-being of clinical nurses.	Cross sectional study	BRS; JCS	Nurses n = 255 73.33% Female Mean age = 31.96 years (SD 7.4)	Clinical nurses who received a COVID- 19 vaccine and those who perceived sufficient staffing in their units reported lower levels of pandemic fatigue. Resilience reduces the effects of pandemic fatigue on clinical nurses’ mental health, sleep quality and job contentment.
Labrague 2020 [67]	COVID-19 Anxiety Among Front-Line Nurses: Predictive Role of Organisational Support, Personal Resilience, and Social Support	Philippines; Non-academic hospitals	To examine the relative influence of personal resilience, social support and organisational support in reducing COVID-19 anxiety in front-line nurses.	Cross sectional study	BRCS; POS; PSSQ	Nurses n = 325 74.8% Female Mean age = 30.94 years (SD 6.67)	Resilient nurses and those who perceived higher organisational and social support were more likely to report lower anxiety related to COVID-19.
Munawar 2020 [68]	Exploring Stress Coping Strategies of Frontline Emergency Health Workers Dealing Covid-19 in Pakistan: A Qualitative Inquiry	Pakistan; Non-academic hospitals	To understand how emergency healthcare workers are dealing with the COVID-19 pandemic, what their stress coping strategies or protective factors are, and the challenges they are facing while dealing with COVID-19 patients.	Qualitative research	Face-to-face or telephone interviews were conducted in a semi-structured format between a HCW and a PhD in Psychology with ample experience in qualitative interviewing. The outline of interview protocols was designed by going through past literature, discussing with experts of qualitative research as well as conducting some pre-interviews with frontline HCWs.	EMS Personnel; Medical Technicians n = 15 0% Female Mean age = 31.87 years (SD 2.82)	Participants practiced and recommended various coping strategies to deal with stress and anxiety emerging from COVID-19 pandemic. Media was reported to be a principal source of raising stress and anxiety among the public. Religious coping as well as their passion to serve humanity and country were the commonly employed coping strategies.
Ong 2016 [71]	The Prevalence of Post-Traumatic Stress Disorder in Intensive Care Unit Staff and the Common Coping Strategies Used	Singapore; Community	To determine the prevalence of PTSD symptoms among ICU staff in Singapore and to determine the common coping strategies employed.	Cross sectional study	Brief-COPE; PTSS-10	Physicians; Nurses n = 107 83% Female Mean age = 31.7 years (SD 7.3)	As caring organizations, institutions have a role in assisting affected employees in dealing with the psychological aftermath of their trauma with sensitivity, understanding and support at every staff level. Despite the high levels of PTSD, the ICU staff had a low rate of negative coping methods.
Ho 2021 [72]	Levels of Burnout and its Association with Resilience and Coping Mechanisms Among Orthopedic Surgery Residents: a Single Institution Experience from Singapore.	Singapore; Academic (University, College)	To describe the levels of burnout and psychological morbidity among orthopedic surgery residents in an ACGME-I accredited program at a single institution. The secondary aim was to determine the associations between levels of burnout and resident characteristics, resilience, and coping mechanisms.	Cross sectional study	Brief-COPE; GRIT-S; RDAS	Residents n = 44 9.1% Female Mean age = 31.4 years (SD 2.3)	Burnout was high in our ACGME-I accredited program. Stressors associated with higher burnout included feeling of inadequate sleep, poor work-life balance, poor relationships with fellow residents/faculty and financial pressures. Residents should be educated on protective coping mechanisms and regular screening to detect burnout should be performed.
Riaz 2021 [73]	Stress and Coping Among Surgery Residents in a Developing Country	Pakistan; Academic (University, College)	To measure the stress level among the surgical residents, identify factors within the learning and work environment that cause stress, and identify different strategies that the residents use habitually to cope with these stresses. The study also explored the relationship between stress levels and continuous assessment scores during residency training.	Cross sectional study	Brief-COPE	Residents n = 68 16.2% Female	Residents in surgical residency programs at AKU perceived themselves to be under stress. Periodic evaluation can help identify residents with high stress levels, and thus specific strategies can be adopted. Academia and hospital administration should join hands in developing interventions to enable residents cope with the situation.

**TABLE 2:** Summary of interventions studies reviewed (Southern and South-eastern Asia, 2016–2021).

Study ID	Title	Country and setting of study	Aim of study	Study design	Intervention used to improve resilience (individual)	Intervention used to improve resilience (systemic)	Population description	Outcome
PRE-COVID								
Kaur 2021 [74]	Mindfulness Integrated Cognitive Behavioural Intervention: Effects on Palliative Cancer Care Professionals	10/25/2021	India; Community	To examine the effects of mindfulness integrated cognitive behavioral interventions (MICBI) for professional care workers at palliative cancer care centers in Bengaluru city of Southern India	Cohort study	Pre-COVID	A single group study design was adopted with pre, post and 3-month follow-up assessment. Measurement points at 1 week before the start of the intervention (pre), 1 week after the last session of the intervention (post), and 3 months after the completion of the intervention (3-month follow-up). The MICBI program included six sessions, occurring weekly for 2–2.5 h. Mindfulness meditation sessions were followed by interactive face-to-face session at the hospice. Participants were asked to practice mindfulness everyday as part of their homework assignments across 6 weeks and beyond. The audio recordings of the guided meditations were given to the participants as homework and also available at the hospice. Outcome variables were professional quality of life (assessed using ProQOL-5) psychological wellbeing (assessed using PWB), and mindfulness (assessed using FFMQ)	Not reported
Kaur 2016 [75]	A Quasi-Experimental Study to Assess the Effect of Relaxation Technique on Stress Related to Adjustment Problems Among Staff Nurses Working in Selected Hospitals of District Jalandhar, Punjab, 2015	5/19/2016	India; Non-academic hospitals	To assess the stress related to adjustment problems among staff nurses of control group and experimental group before the intervention and compare after intervention	Case control study	Pre-COVID	Progressive muscle relaxation is a systematic technique for achieving a deep state of relaxation. It was developed by Chicago physician Edmond Jacobson who discovered that a muscle could be relaxed by first tensing muscles for a few seconds and then releasing it. Dr. Jacobson found that tensing and relaxing various muscle groups throughout the body produces a deep state of relaxation capable of relieving a variety of conditions	Not reported
Mandal 2021 [76]	Effect of Structured Yoga Program on Stress and Professional Quality of Life Among Nursing Staff in a Tertiary Care Hospital of Delhi - A Small Scale Phase-II Trial	01/09/2021	India; Community	To study the efficacy of structured yoga on stress and the professional quality of life among nursing staff. Also to assess the sustainability of this yoga program among this nursing staff of a tertiary care hospital in Delhi	Randomized controlled trial	Pre-COVID	The participants were allocated to 2 groups - intervention (i.e., yoga group) or the wait-listed group. The yoga module (consisting of asana, pranayama, and deep relaxation technique) was developed by a committee of yoga physicians and yoga therapists at the institutional yoga facility. They adopted the 5 min deep relaxation technique practiced in supine position, Shavasana (Corpse Pose), an evidence-based way to completely relax the whole body within a short amount of time. Two 50-min sessions were conducted weekly for 12 consecutive weeks. A minimum of 20 sessions in the 12 weeks period was considered completed intervention. Perceived stress was assessed using the PSS-10 and morning serum cortisol level. Professional quality of life was assessed using ProQOL-5 scale. Other non-specific outcome of stress was serum high sensitive C-reactive protein (HSCRP), systolic, and diastolic blood pressure	Not reported
Suyi 2017 [77]	Effectiveness of Mindfulness Training in Reducing Stress and Burnout for Mental Health Professionals in Singapore	10/01/2017	Singapore; Academic (University, College)	To examine the effectiveness of a mindfulness program in increasing mindfulness and compassion, and reducing stress and burnout, among mental health professionals in Singapore	Non-randomized experimental study	Pre-COVID	Participants attended six sessions of mindfulness training, offered once a week. The participants were evaluated at three points using the same assessments: pre-intervention (time 1), post-intervention (time 2), and 3 months after the intervention (time 3). Measures used include FFMQ, SCM-SF, CS, PSS-10, and OLBI.	Not reported

These studies were further categorized into main themes: fatigue, stress, burnout, satisfaction, and resilience.

### Methods Used to Assess Fatigue, Stress, Burnout, Satisfaction, and Resilience

#### Fatigue

Two studies, both conducted in the Philippines during the COVID-19 pandemic, evaluated fatigue [9, 66]. They aimed to examine the role of resilience in mitigating the negative effects of pandemic-related stressors on nurses’ wellbeing and job outcomes. The first study focused on pandemic fatigue and its impact on clinical nurses’ mental health, sleep quality, and job contentment [9]. The second study investigated the relationship between compassion fatigue, job outcomes (satisfaction and turnover intention), and care quality among frontline nurses [66].

Both studies employed cross-sectional designs and used various scales to measure resilience, including the BRS and the Brief Resilience Coping Scale (BRCS). The outcomes of these studies consistently highlighted the protective role of resilience in the face of pandemic-related challenges. The first study found that resilience reduces the effects of pandemic fatigue on nurses’ mental health, sleep quality, and job contentment, with vaccinated nurses and those perceiving sufficient staffing reporting lower levels of fatigue [9]. The second study revealed that psychological resilience diminishes the negative impact of compassion fatigue on job satisfaction, turnover intention, and quality of care [66].

These findings underscore the importance of implementing resilience-promoting interventions and proactive measures to reduce fatigue and support nurses’ wellbeing. Both studies emphasize the need for nursing administrators to prioritize strategies that enhance resilience, as this can lead to improved job satisfaction, better retention rates, and higher quality of care in healthcare settings, particularly during challenging times such as a pandemic [9, 66].

#### Stress

The scale that was used most to measure stress was the PSS-10. This scale was used to assess stress and its effect on coping strategies and burnout. These studies were conducted among various healthcare professionals (including mental health workers, residents in training, nurses, and surgical residents) in different Southern and Souther-eastern Asian contexts (Singapore, Malaysia, and Pakistan), consistently finding that mindfulness, active coping strategies, and targeted interventions can help mitigate stress and improve mental health outcomes in these high-pressure medical environments. [25, 48, 51, 73].

Other methods to measure stress included individual interviews [32, 34] and the Generalized Anxiety Disorder 7-item (GAD-7) [45, 47], Depression Anxiety Stress Scale (DASS-21) [46], Kessler Psychological Distress Scale (K-10) [44], Secondary Traumatic Stress Scale (STSS) [54], State-Trait Anxiety Inventory (STAI) [61], and a modified instrument used during MERS-CoV [49, 63]. These studies consistently found high levels of stress and anxiety among healthcare workers, with contributing factors including fear of infection, perceived stigma, and heavy workloads. However, they also identified resilience as a significant protective factor. Outcomes emphasized the urgent need for psychological support and interventions to enhance resilience among healthcare workers. Recommendations included implementing programs to strengthen resilience, providing opportunities for stress reduction, and ensuring organizational support. The findings highlight the importance of addressing the mental health of healthcare professionals to maintain their wellbeing and ensure effective patient care, particularly during challenging times such as the COVID-19 pandemic [32, 34, 44–47, 49, 54, 61, 63].

#### Burnout

The Maslach Burnout Inventory Human Services Survey (MBI-HSS) was used in all the studies to measure burnout. These studies, conducted across various Southern and South-eastern Asian countries including Pakistan, India, Singapore, and Indonesia, aimed to assess burnout levels and associated factors among diverse healthcare professionals, ranging from physicians and residents to nurses and pharmacy technicians. The research collectively sought to identify prevalence rates of burnout, explore its relationship with factors such as workplace violence, resilience, coping strategies, and demographics, and in some cases, evaluate the effectiveness of burnout screening tools. Outcomes consistently revealed high levels of burnout across different healthcare specialties, with contributing factors including workload, inadequate sleep, poor work-life balance, and financial pressures. The studies emphasized the need for interventions to enhance resilience, promote effective coping mechanisms, and improve work environments. Recommendations included regular burnout screening, education on protective coping strategies, and organizational efforts to mitigate workplace stressors. Overall, these findings underscore the critical importance of addressing burnout in healthcare settings to maintain the wellbeing of professionals and ensure quality patient care [26, 35, 36, 41, 53, 62, 71, 72].

#### Satisfaction

There were only a couple of studies which examined job satisfaction and its influencing factors among psychiatric nurses in Singapore’s only tertiary psychiatric institution, finding a positive correlation between job satisfaction and resilience, with nurses generally satisfied but with room for improvement through enhancing personal resilience [24]. The other study by Achour et al. used an eight-item survey developed by Hackman and Oldham in 1975 and recommended different stress management interventions for older and younger nurses who had the least satisfaction [31].

#### Resilience

**FIGURE 3:**
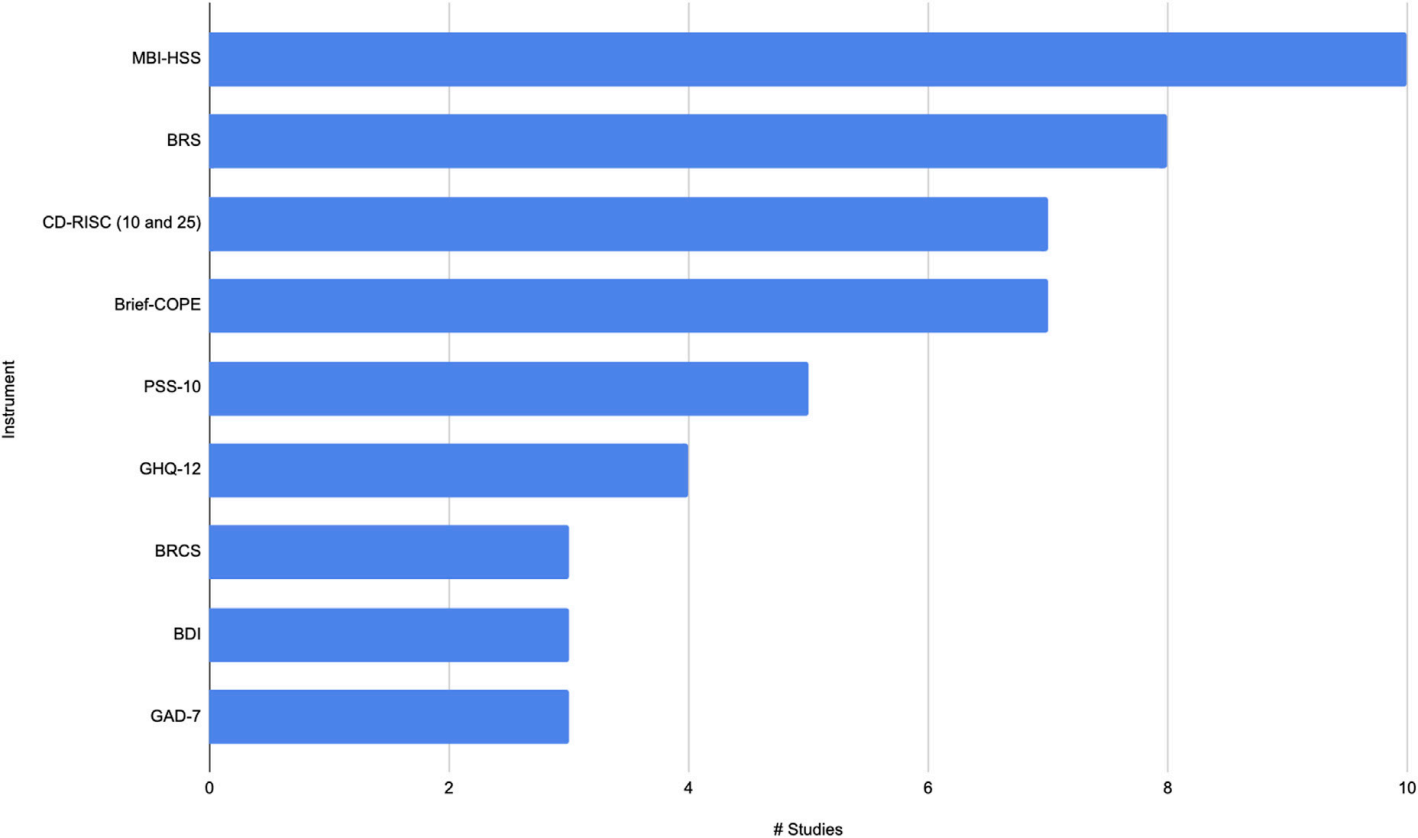
Most used instruments/scales/surveys (Southern and South-eastern Asia, 2016–2021).

Of the 51 studies that identified methods used to assess burnout, fatigue, stress, or resilience, only four studies did not report any scales to measure resilience. Several studies used certain scales to measure resilience. Frequently used scales were BRS (eight studies) [9, 33, 41, 44, 45, 47, 54, 60], Brief-COPE (seven studies) [48, 51, 62, 64, 71–73], CD-RISC-25 (five studies) [37, 46, 53, 55, 61], CD-RISC-10 (two studies) [39, 64], and BRCS (three studies) (Figure 3) [56, 66, 67].

The eight studies that used BRS were published during the COVID-19 pandemic and were done in academic and community settings, in Bangladesh, India, Pakistan, Philippines and Singapore. These studies assessed resilience using BRS in nurses, physicians, pharmacists, and other HCP. Authors of various studies highlighted the need to develop resilience of HCP including providing support through organizational efforts. Of note, the study by Labrague 2021, noted that nurses who received the COVID-19 vaccine and had “perceived sufficient staffing in their units reported lower levels of pandemic fatigue” [9].

The Brief-COPE (Carver 1997) was used in seven studies [15]. The studies were published predominantly during the COVID-19 pandemic with one study published pre-pandemic in 2016. Some of the studies also used other instruments in combination with Brief-COPE. These studies were done in Indonesia, Malaysia, Pakistan and Singapore, in both academic and community settings and included nurses, physicians and residents. The authors of these studies noted stressors including inadequate sleep, significant work hours as contributing to burnout and studies provided solutions such as engaging in sports, “seeking social support, positive thinking” as “active coping strategies” [48, 51, 62, 64, 71–73].

The CD-RISC-25 is a 25-item self-rating tool, developed by Kathryn M. Connor and Jonathan R.T. Davidson in 2003 [14]. The CD-RISC measures various aspects of resilience such as adaptability, control under pressure, and personal strength. Individuals with higher CD-RISC scores typically demonstrate better coping mechanisms and are better equipped to handle stress and recover from setbacks [78].

The studies using CD-RISC-25 and CD-RISC were done in Malaysia, India, Indonesia and Singapore. Nurses, pharmacists, medical technicians, social workers and residents (trainees) participated in these studies from both academic and non-academic settings. Setiawati 2021 et al noted an inverse correlation of level of resilience and anxiety of HCP [61]. Other studies summarized the need for “effective interventions for improving resilience” and the need to support frontline staff by organizations, with social support [37, 46, 53, 55].

During the COVID-19 period, three studies, all done in the Philippines, reported using the BRCS (Sinclair and Walston 2004) [79]. These three studies evaluated nurses (predominantly females - 74.5%–74.8%) perceptions of discrimination and resilience during COVID-19. These studies advocated for the need for nurses to be supported and to develop resilience [56, 66, 67].

Some studies also used resilience assessments using questionnaires related to religious beliefs and practices and one study used the Brief Religious Coping Scale (BRCOPE) (Pargament 1997) [80].

### Interventions Used to Build Resilience

Four intervention studies involving 172 participants were conducted to enhance individual resilience among healthcare workers. The studies varied in design: one cohort, one case-control, one randomized controlled trial, and one non-randomized controlled trial. They targeted community settings, non-academic hospitals, and an academic university but did not address systemic resilience [74–77].

The studies employed diverse interventions: Mindfulness-Integrated Cognitive Behavioral Therapy (MiCBT). This combines Cognitive Behavioral Therapy with mindfulness practices for a holistic approach. Progressive Muscle Relaxation, developed by Dr. Jacobson, is a technique involving tensing and relaxing muscle groups to achieve deep relaxation and alleviate stress. Yoga integrates physical postures, breath control, meditation, and ethical principles to enhance overall wellbeing [74–77].

Participants included healthcare professionals such as staff nurses, palliative cancer care professionals, and mental health professionals. The studies were primarily conducted in India, with one in Singapore. Most participants were female (68%–94%), with a significant representation of those under 40-years old. A possible reason for the high female representation could be the high rate of nurse recruitment. Age reporting varied across studies, while Kaur 2021 and Mandal 2021 reported average age, the other studies mentioned the percentage of the age range.

The findings indicate that these interventions can positively influence resilience and reduce stress among healthcare workers, though effectiveness may differ by population and context. Notably, Suyi’s 2017 study focused on mental health professionals; while stress levels decreased, burnout did not show a similar reduction [77].

The interventions demonstrate promise for improving individual resilience, highlighting the need for further research to explore their effectiveness across different healthcare contexts and populations.

## Discussion

HCP have experienced burnout even pre-pandemic. However, very early on with the start of the COVID-19 pandemic, strategies to develop resilience of HCP were emphasized [81]. This study is a scoping review and not a meta-analysis because of the lack of uniformity and varied data. However, we have identified certain key aspects of assessing and developing individual resilience as noted below.

### Mindfulness

The APA defines mindfulness as “awareness of one’s internal states and surroundings” [82]. Many mindfulness interventions have been practiced, including mindfulness-based cognitive behavior therapy, mindfulness-based stress reduction (MBSR), and mindfulness meditation. MBSR was originally developed by Professor Jon Kabat-Zinn as a tool combining mindfulness meditation, yoga, and body awareness strategies to help individuals become more aware of their surroundings and attune to their present sensations and feelings to understand and relieve present stress and anxiety [83].

Two of the five studies exploring interventions to improve individual resilience utilized mindfulness-based interventions [74, 77]. Kaur’s study, conducted in India, defines mindfulness through the Pali word “Vipassana,” meaning “to observe in a special way,” or being present “here and now.” The study aimed to evaluate the effects of mindfulness integrated cognitive behavioral interventions (MICBI) on HCP to improve compassion satisfaction, reduce secondary traumatic stress, decrease workload, enhance overall wellbeing, and improve mindfulness skills. Kaur developed a unique MICBI program based on a comprehensive literature review, comprising six separate two-hour weekly sessions of mindfulness meditation, followed by interactive face-to-face discussions, and daily homework assignments, including guided meditations [74].

Suyi’s study, conducted in Singapore, does not explicitly define mindfulness; however, it highlights the beneficial impact of mindfulness training in reducing stress and burnout while improving attitudes of HCP. The study examined the effectiveness of a modified MBSR program in enhancing mindfulness and compassion while reducing stress and burnout among HCP. The traditional 8-week MBSR course was shorted to a 6-week program [77]. This condensed format proved to be equally effective as the standard MSBR program [77, 84].

A scoping review by Kriakous et al. [84], which included 30 studies conducted primarily in North America and Europe, confirmed that MBSR effectively reduces anxiety, depression, and stress experienced by HCP, while also increasing individual mindfulness and self-compassion. However, the review noted that MBSR programming did not significantly reduce burnout or improve resilience amongst HCP [84]. Similarly, Suyi [77] found that while MBSR led to reductions in stress and improvement in mindfulness and compassion among HCP, it did not impact burnout levels [77]. In contrast, Kaur’s (2021) MICBI program demonstrated significant reductions in both stress and burnout, as well as improvements in compassion satisfaction, wellbeing, and mindfulness skills after 3 months [74]. This difference may be attributed to the daily individual mindfulness practices, such as meditation, which were integral to the MICBI program. Daily practice was associated with enhanced psychological function and outlook [84].

### CD-RISC Score Interpretation

CD-RISC scores are interpreted based on populations and geographic settings [78]. In cross-cultural comparisons, higher or lower average scores might reflect different cultural approaches to stress and coping, rather than actual differences in resilience levels. For example, in the initial validation study by Connor and Davidson, the mean score among the general US population was 80.4, indicating a high resilience [14]. However, a study conducted by Yu and Zhang (2007) with Chinese participants (including those from Hong Kong) found a mean CD-RISC score of 52.6 [85]. This study confirmed the scale’s reliability but also noted that cultural attitudes toward self-reporting can lead to differences in scores. The US culture emphasizes individualism and personal achievement, which may encourage individuals to report higher levels of personal resilience. There is a cultural acceptance of acknowledging personal strengths and coping abilities, potentially leading to higher self-reported resilience.

On the other hand, Hong Kong’s culture places a strong emphasis on collectivism and interdependence, which may influence individuals to be more modest when self-reporting resilience [86].

The CD-RISC is a robust tool for assessing resilience across cultures, but interpretation of scores must account for cultural differences. A lower score in Hong Kong does not necessarily indicate less resilience but may reflect different cultural expressions of resilience. This difference in the interpretation of CD-RISC scores across various countries highlights that resilience-building programs should also be culturally implemented. In Hong Kong, interventions might focus on enhancing family support and community connections, whereas in the US, fostering individual coping strategies may be more effective.

### Individual Versus Health Systems Resilience

This scoping review focused specifically on individual resilience, rather than health systems resilience (population health of system). Individual psychological resilience refers to the ability of HCP to adapt and thrive despite the stressors and challenges they face. It includes emotional regulation, coping strategies, and the ability to recover from stress while continuing to provide effective care. Studies have shown that higher individual resilience is associated with lower levels of stress, anxiety, and burnout among healthcare workers. For instance, Huffman et al. found that higher resilience was linked to lower stress, anxiety, fatigue, and sleep disturbances among healthcare providers during the pandemic [87]. Similarly, Fleuren et al. demonstrated that personal resilience negatively correlates with depressive complaints and worries about infections [88].

Health system resilience refers to the capacity of health systems to prepare for, respond to, and recover from crises while maintaining core functions and learning from these events to improve future responses [89–91]. This concept encompasses both the ability to handle known shocks and the intrinsic flexibility to adapt to unpredictable events [92, 93].

The two forms of resilience are interdependent as they both aim to reduce burnout among healthcare personnel. As shown by Shanafelt et al., individual psychological resilience directly impacts the functioning of healthcare systems, particularly during crises. Similarly, a well-functioning health system provides the support necessary for healthcare workers to maintain their resilience [94].

Interestingly, Burau et al. [95] found that when investigating health system resilience with a particular focus on the health workforce, the health workforce seemingly had little impact on the health system during the first wave of the COVID-19 pandemic [95]. However, a recent UK-based study by Martin et al. (2024) found that nearly half of HCP considered or acted on leaving the healthcare workforce, linked to higher burnout scores and mental health issues like depression and anxiety [96]. This does indicate that further studies are needed to understand the complex relationships of resilience of individual HCP and the resilience of health systems they work in, especially with regards to the COVID-19 pandemic given the fact that we know both individual and health systems resilience do work hand in hand in response to health crises, such as the COVID-19 pandemic.

**FIGURE 4:**
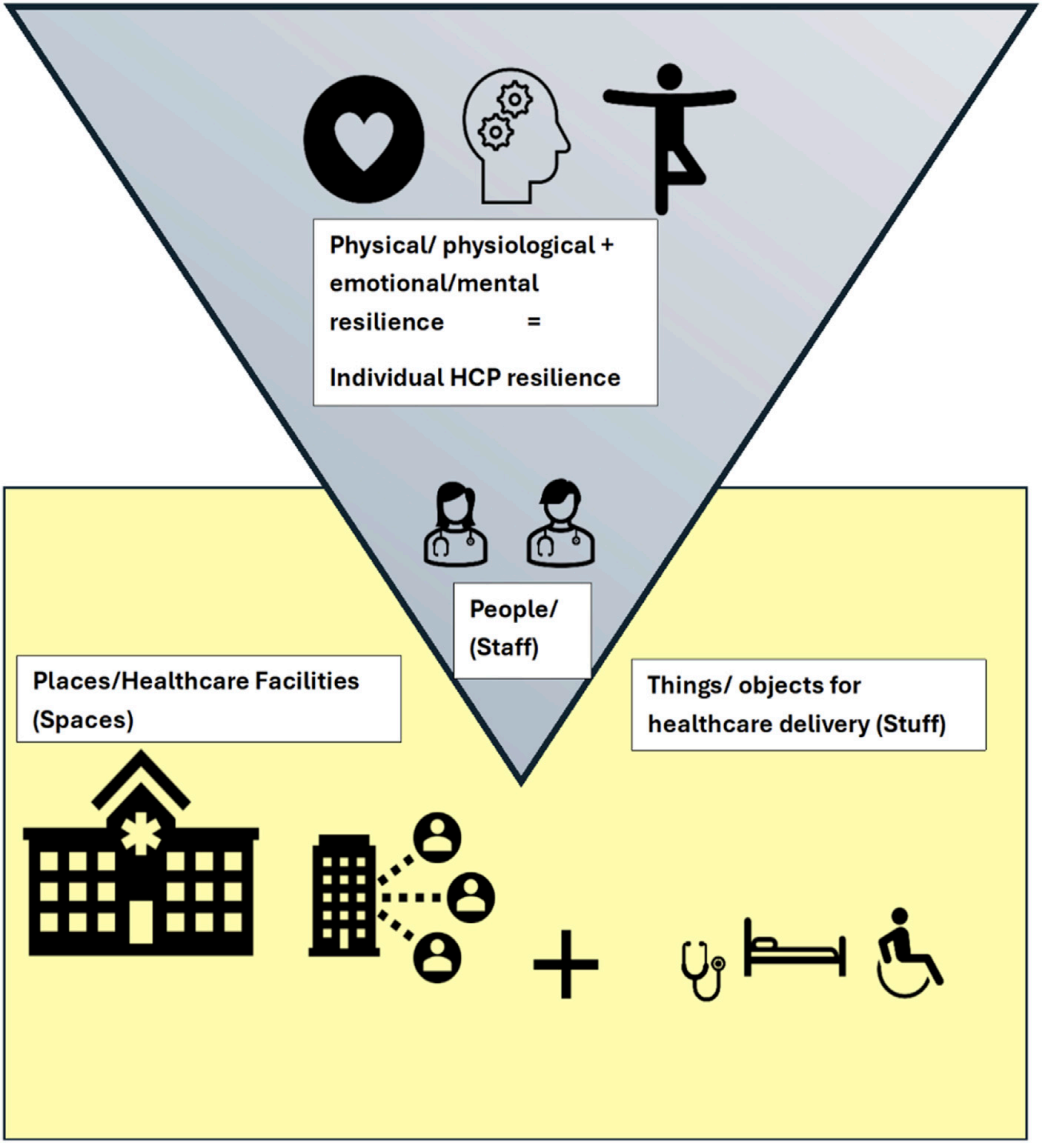
Overview of components of health systems resilience (Mathew, USA, 2024).

When comparing the two forms of resilience, individual resilience focuses on the personal capacity of healthcare workers to adapt to stress and trauma, while system resilience pertains to the broader infrastructure’s capacity to respond to emergencies. Individual-level resilience is developed through internal coping mechanisms and is crucial to the overall health system resilience and response. Interventions for building psychological resilience often involve personal development programs such as mindfulness training, cognitive-behavioral strategies, and stress management programs [97]. In contrast, health system resilience is bolstered by governance reforms, resource allocation, and systemic policies that improve the capacity of the workforce and infrastructure to handle crises, as noted by Kruk et al [91, 98]. Figure 4 illustrates how individual-level resilience fits within a broader health systems resilience framework.

### Limitations

This study has several limitations that must be considered. First, the review was restricted to articles published in English. Additionally, the study focused exclusively on individual psychological resilience among HCP and did not address resilience at the system level (facility or hospital). Moreover, individual physiological resilience, such as the presence of comorbidities or physical health factors, was not considered in this analysis. Another limitation that became clear during data analysis is that while the CD-RISC has been shown to be a robust tool for assessment of resilience across cultures, the interpretation of results requires knowledge of the countries’ cultural context. This may limit comparison of data across multiple countries given the assumption that cultural context informs an individual’s responses on the CD-RISC tool. Furthermore, a formal analysis of study quality was not conducted; therefore, results are summarized without weighing for the risks of bias. Finally, the initial search of publications for this scoping review was concluded in December 2021. We acknowledge other studies may have been published since the initial search and further analysis will be needed to assess resilience of HCP. Future research should examine psychological resilience among HCP in more diverse settings outside of Southern and South-eastern Asia to enhance understanding across different healthcare and cultural environments.

### Conclusion

This scoping review of publications from 2016 to 2022 highlights the extensive body of research conducted across Southern and South-eastern Asia to assess burnout, fatigue, stress, and resilience amongst HCP. Several widely used and validated tools, including BRS, CD-RISC, and Brief-COPE, have been employed to measure resilience. The CD-RISC is notable for its variability in interpretation across different populations and settings. Moving forward, further studies using the CD-RISC should consider the demographic and geographic context of their subjects to ensure more accurate and meaningful results.

This review underscores the pressing need for effective resilience-building interventions at both individual and systemic levels. The findings emphasize the critical role of resilience in mitigating pandemic-related challenges, such as job satisfaction, HCP mental health, and overall quality of patient care. By providing an overview of some of the scales used to measure these factors, this scoping review offers valuable insights into the mental health of Southern and South-eastern Asia’s healthcare workforce, both before and after the COVID-19 pandemic. As we continue to face increasing stressors—ranging from emerging pathogens to environmental changes and natural disasters—building resilience among healthcare professionals will become increasingly essential to maintaining both their wellbeing and the quality of care they provide.
