# Supplementary material for: Exploring Stress, Fatigue, Burnout, and Resilience Among Healthcare Personnel in Southern and South-Eastern Asia: A Scoping Review
Source: Public Health Rev. 2025 Nov 26;46:1608603. doi: 10.3389/phrs.2025.1608603 (PMC12690366; doi:10.3389/phrs.2025.1608603)
Supplement: Supplementary file 1 [file Table1.docx]

6-D - Six-Dimension Scale of Nursing Performance Scale (Schwirian 1978) [102]

52-item questionnaire measuring nurse behaviors grouped into six performance subscales: leadership (5 items), critical care (7 items), teaching/collaboration (11 items), planning/evaluation (7 items), interpersonal relations/communications (12 items), and professional development (10 items).

ABQ - Avoidance Behavior Questionnaire (Marjanovic et al. 2007) [103]

6-item questionnaire measuring avoidant qualities such as minimizing direct contact with patients, missing work, and refusing patient assignments. Items are rated using a 4-point Likert scale running from 1 (‘not at all’) to 4 (‘a great deal’).

AITCS - Assessment of Interprofessional Team Collaboration Scale (Orchard et al. 2012) [104]

47-item questionnaire measuring self-reported level of collaboration amongst professionals. It consists of four subscales: partnership, cooperation, coordination, and shared decision making). Items are rated on a 5-point Likert scale ranging from 1 (‘never’) to 5 (‘always’).

APRQ - Adult Personal Resilience Questionnaire (Taormina 2015) [105]

30-item questionnaire targeting six dimensions of adult resilience, with five items in each dimension: determination, endurance, adaptability, recuperability, comfort zone, and life calling). Items are rated using a 5-point Likert scale ranging from 1 (‘strongly disagree’) to 5 (‘strongly agree’).

AS - Attitude Scale (Su et al. 2007) [106]

13-item questionnaire measuring three constructs in evaluating the changes in attitude towards the SARS outbreak in SARS unit subjects: knowledge and understanding of SARS, perceived negative feelings towards SARS, and positive attitudes towards caring for SARS patients. Items are rated on a 4-point Likert scale ranging from 0 (‘Strongly disagree’) to 3 (‘Strongly agree’).

BAI - Beck Anxiety Inventory (Beck and Steer 1988) [107]

21-item questionnaire measuring the intensity of physical and cognitive anxiety symptoms during the past week. Items are rated using a 4-point Likert scale.

BDI - Beck Depression Inventory (Beck, et al. 1961) [108]

21-item questionnaire measuring characteristic attitude and symptoms of depression. Items are rated on a 4-item Likert scale.

BRCOPE - Brief Religious Coping Scale (Pargament 1997) [80]

14-item questionnaire assessing two sub-scales of religious coping: positive (PCOPE) and negative coping (NCOPE). Positive coping reflects a positive relationship with the Almighty in times of distress. On the other hand, negative coping refers to interpersonal struggles and conflict with the omnipotent presence of God in challenging times. Items are rated using a 4-point Likert scale ranging from 0 (‘not at all’) to 3 (‘a great deal’).

BRCS - Brief Resilient Coping Scale (Sinclair & Walston 2004) [7]

4-item questionnaire measuring resilience. Items are rated using a 5-point Likert scale ranging from 0 (‘does not describe me at all’) to 5 (‘describes me very well’).

Brief-COPE - Brief Coping Orientation to Problems Experienced (Carver 1997) [15]

28-item questionnaire used to assess the type and frequency of coping strategies employed by residents. It comprises self-interruption, active adapting, substance use, use of passionate help, venting, positive reframing, humor, acceptance, religion, and self-fault. Items are rated using a 4-point Likert scale ranging from 1 (‘never’) to 4 (‘very often’).

BRS - Brief Resilience Scale (Smith et al. 2008) [3]

6-item questionnaire measuring the ability of individuals to bounce back or recover from stress. Items are rated on a 5-point Likert scale ranging from 1 (‘strongly disagree’) to 5 (‘Strongly agree’).

BSCS - Brief Self Control Scale (Molouf et al. 2014) [109]

13-item questionnaire measuring self-control. Items are rated using a 5-point Likert scale ranging from 1 (‘not at all like me’) to 5 (‘very much like me’).

BSSC - Brief Scale for Social Support (Moser et al. 2012) [110]

8-item questionnaire measuring emotional, interpersonal and material support. Items are rated using a 5-point Likert scale ranging from 1 (‘almost none’) to 5 (‘very much’).

CAS - COVID-19 Anxiety Scale (Lee 2020) [111]

5-item questionnaire measuring dysfunctional anxiety associated with the coronavirus crisis experienced by an individual in the past two weeks. Items are rated on a 5-point Likert scale ranging from 0 (‘not at all’) to 4 (‘nearly every day’).

CAD - COVID-19-Associated Discrimination scale (Liu et al. 2020) [112]

10-item questionnaire measuring nurses’ experiences of discriminatory behavior from the public since the onset of the COVID-19 pandemic. Items are answerable by a response of ‘yes’, ‘no’, or ‘unsure’.

CD-RISC-10 - Connor–Davidson Resilience Scale (Campbell-Sills & Stein 2007) [113]

10-item self-rating tool measuring resilience traits. Items are rated on a 5-item Likert scale ranging from 0 (‘never) to 4 (‘almost always’).The scores on each item are summed up for the final score, with higher scores representing a higher level of resilience. “High” resilience is defined by scoring in the higher 70th percentile. “Low” resilience is defined by scoring in the lower 30th percentile.

CD-RISC-25 - Connor–Davidson Resilience Scale (Connor & Davidson 2003) [14]

25-item self-rating tool measuring the ability to cope with stress and adversity with four aspects of resilience: hardiness (8 items), optimism (6 items), resourcefulness/self-efficacy (8 items), and meaningfulness/purpose (3 items). Items are rated on a 5-item Likert scale ranging from 0 (‘never’) to 4 (‘almost always’). Higher scores suggest higher resilience.

CFS - Compassion Fatigue Scale (Figley 1995, short form) [114]

13-item questionnaire measuring CF with five items assessing secondary trauma and eight items assessing job burnout. Items are rated using a 10-point Likert scale ranging from 1 (‘never’) to 10 (‘very frequent’).

CJSSQ - Consultants Job Stress and Satisfaction Questionnaire (Ramirez et al. 1996) [115]

46-item questionnaire measuring job stress and satisfaction. One question asked “Overall, how stressful/satisfying do you find your work?" on a scale of 0 (‘not at all’) to 4 (‘extremely’). The questionnaire also includes 25 specific sources of stress and 17 sources of satisfaction. Each source of stress/satisfaction was rated according to the extent it had contributed to the overall stress or satisfaction experienced at work during the previous few months, on a scale of 0 (‘not at all’) to 3 (‘a lot’).

CS - Compassion Scale (Pommier 2010) [116]

24-item self-report questionnaire measuring compassion for others. It consists of both positively and negatively worded items. Items are rated using a 5-point Likert scale ranging from 1 (‘almost never’) to 5 (‘almost always’)

DASS-21 - Depression Anxiety Stress Scale (Lovibond 1995, short form) [117]

21-item (short version) of a 42-item self-report instrument measuring three related negative emotional states: depression, anxiety and tension/stress. Consists of seven items for each of the three psychological distress components (dysfunction, distress, and deviance). Items are rated using a 4-point Likert scale ranging from 0 (‘did not apply to me at all’) to 3 (‘applied to me very much, or most of the time).

DRS-15 - Dispositional Resilience Scale (Bartone 2007) [118]

15-item questionnaire measuring psychological hardiness, covering aspects related to commitment, control, and challenge. Items are rated using a 4-point Likert scale ranging from 0 (‘not at all true’) to 3 (‘completely true’).

DTS - Davidson trauma scale (Davidson 1997) [119]

17-item self-report measuring the 17 DSM-IV symptoms of PTSD. Items are rated using a 5-point Likert scale ranging from 0 (‘not at all’) to 4 (‘every day') and severity scales ranging from 0 (‘not at all distressing’) to 4 (‘extremely distressing’). Respondents are asked to identify the trauma that is most disturbing to them and to rate, in the past week, how much trouble they have had with each symptom.

DUREL - Duke University Religion Index (Koenig et al. 1997) [120]

5-item questionnaire assessing the three major dimensions of religiosity: organizational religious activity, non-organizational religious activity, and intrinsic religiosity (or subjective religiosity).

FAA - Fear and Anxiety Assessment (Ahmed et al. 2020) [121]

22-item questionnaire measuring fear and anxiety levels of health care professionals toward COVID-19. Responses are ‘yes’, ‘no’, or ‘unsure’.

FFMQ - Five Facets Mindfulness Questionnaire (Baer et al. 2008) [122]

39-item self-reported questionnaire measuring the five facets of mindfulness: observing, describing, acting with awareness, non-judging, and non-reactivity to inner experience. Items are rated using a 5-point Likert scale ranging from 1 (‘never or very rarely true’) to 5 (‘very often or always true’).

GAD-7 - Generalized Anxiety Disorder Scale (Spitzer et al. 2006) [123]

7-item questionnaire measuring severity of anxiety in the last two weeks. Items are rated using a 4-point Likert scale ranging from 0 (‘not at all’) to 3 (‘nearly every day’).

GHQ-12 - General Health Questionnaire-12 (Goldberg & Williams 1988) [124]

12-item questionnaire measuring psychiatric morbidity. Items are rated using a 4-point Likert-like scale ranging from 1 (‘hardly ever’) to 5 (‘almost always’).

GRIT-S - Short Grit Scale (Duckworth & Quinn 2009) [125]

8-item questionnaire measuring participants’ trait-level perseverance and passion for long-term goals, as a reflection of resilience. Items are rated using a 5-point Likert scale ranging from 1 (‘not like me at all’) to 5 (‘very much like me’).

GQ - Gratitude Questionnaire (McCullough et al. 2002) [126]

6-item questionnaire designed to assess individual differences in the proneness to experiencing gratitude in daily life. Items are rated using a 7-point Likert scale ranging from 1 (‘strongly disagree’) to 7 (‘strongly agree’).

GSE-6 - General Self Efficacy Scale Short Form (Romppel et al. 2013) [127]

6-item inventory measuring self-efficacy beliefs within an individual. Items are rated using a 4-point Likert scale ranging from 1 (‘not at all true’) to 4 (‘exactly true’).

HADS - Hospital Anxiety and Depression Scale (Zigmond & Snaith 1983) [16]

14-item questionnaire consisting of two subscales that evaluate symptoms of depression and anxiety. Items are rated using a 4-point Likert scale. A score of 8 or greater is suggestive of the possible presence of anxiety or depression.

HIV-SS-12 - HIV Stigma Scale Version 12 (Reinius et al. 2017) [128]

12-item questionnaire measuring perceived stigma towards HIV. It contains four subscales containing three items each: personalized stigma, disclosure concerns, concerns with public attitudes, and negative self-image. Items rated using a 4-point Likert scale ranging from 1 (‘strongly agree’) to 4 (‘strongly disagree’).

HWSS - Healthcare Workers Stigma Scale (Chew 2020) [51]

12-item questionnaire adapted from the HIV Stigma Scale used to assess residents’ level of perceived stigma. Items can be grouped into four subscales: personalized stigma, disclosure concerns, concerns about public attitudes, and negative self-image. Items rated using a 4-point Likert scale ranging from 1 (‘strongly disagree’) to 4 (‘strongly agree’).

IES-R - Impact of Events Scale-Revised (Weiss & Marmar 1997) [129]

22-item questionnaire measuring subjective distress caused by traumatic events. Items are rated using a 5-point Likert scale ranging from 0 (‘never’) to 4 (‘very often’).

ISI - Insomnia Severity Index (Morin 1993) [130]

7-item screening tool assessing more insomnia disorders. Items are rated using a 5-point Likert scale.

JCS - Job Contentment Scale (Taunton et al. 2004) [131]

7-item questionnaire measuring job contentment in nurses during the coronavirus pandemic. Items are rated using a 5-point Likert scale ranging from 1 (‘strongly disagree’) to 5 (‘strongly agree’).

JSPE - Jefferson Scale of Physician Empathy (Hojat 2001) [132]

20-item questionnaire measuring empathy in the context of health professions education and patient care. Items are rated using a 7-point Likert scale ranging from 1 (‘strongly disagree’) to 7 (‘strongly agree’).

JSS - Job Satisfaction Scale (Taunton et al. 2004) [131]

7-item questionnaire measuring nurses' satisfaction with their present job during the height of the coronavirus crisis. Items are rated using a 5-point Likert scale ranging from 1 (‘strongly disagree’) to 5 (‘strongly agree’).

K-10 - Kessler Psychological Distress Scale (Kessler et al. 2002) [133]

10-item questionnaire measuring levels of psychological distress. Items are rated using a 5-point Likert scale ranging from 1 (‘none of the time’) to 5 (‘all of the time’).

MBI-HSS - Maslach Burnout Inventory Human Services Survey (Maslach & Jackson 1981) [11]

22-item questionnaire assessing the three dimensions of burnout: emotional exhaustion (9 items), depersonalization (5 items), and reduced personal accomplishment (8 items) used to measure burnout in healthcare personnel. Items are rated using a 7-point Likert-like scale ranging from 0 (‘never’) to 6 (‘everyday’).

MBI-GS - Maslach Burnout Inventory-General Survey (Maslach et al. 1996) [134]

16-item questionnaire with a total score from three dimensions, namely, exhaustion, cynicism, and professional efficacy. Items are rated using a 7-point Likert scale.

MDI - Major Depression Inventory (Bech et al. 2001) [135]

10-item questionnaire measuring the severity of depressive states and diagnosing depression. Items are rated using a 6-point Likert scale ranging from 0 (‘at no time’) to 5 (‘all of the time’).

MHI - Mental Health Inventory (Veit & Ware 1983) [136]

5-item questionnaire measuring frontline nurses’ mental health status during the coronavirus pandemic. Items are rated using a 6-point Likert scale ranging from 0 (‘none of the time’) to 5 (‘all of the time’).

MMSS - McCloskey and Mueller Satisfaction Scale (Mueller & McCloskey 1990) [137]

31-item questionnaire measuring job satisfaction among nurses consisting of eight subscales: satisfaction with extrinsic rewards, scheduling, family/work balance, co-workers, interaction, professional opportunities, praise/recognition, and control/responsibility. Items are rated using a 5-point Likert scale ranging from 1 (‘very dissatisfied’) to 5 (‘very satisfied’).

MOS-SS - Medical Outcomes Study Social Support Survey (Ware & Sherbourne 1991) [138]

19-item social support survey measuring functional social support in community dwelling chronically ill persons. The 19 items cover four domains (emotional/informational support, tangible/instrumental support, positive social interaction, and affection) recommended for both combined and individual use. Items are rated using a 5-point Likert scale ranging from 1 (‘none of the time’) to 5 (‘all of the time’).

NAQ - Negative Acts Questionnaire (Einarsen et al. 2009) [139]

22-item questionnaire measuring exposure to bullying in the workplace covering three underlying factors: personal bullying, work-related bullying, and physically intimidating forms of bullying. Items are rated using a 5-point Likert scale ranging from 1 (‘never’) to 5 (‘daily’).

OLBI - Oldenburg burnout inventory (Demerouti 1999) [140]

16-item self-report questionnaire measuring burnout and consisting of two subscales: exhaustion and disengagement from work. Items are rated using a 4-point Likert scale ranging from 1 (‘strongly agree’) to 4 (‘strongly disagree’).

PCL-C - Civilian version of PTSD Checklist (Weathers et al. 1994) [141]

17-item self-report measure that corresponds to the Diagnostic and Statistical Manual of Mental Disorders, 4th edition symptoms of PTSD. Items are rated using a 5-point Likert scale ranging from 1 (‘not at all’) to 5 (‘extremely’).

PCQ - Psychological Capital Questionnaire (Luthans et al. 2006) [142]

24-item questionnaire measuring how respondents feel about themselves at the moment. It consists of four factors of psychological capital: self-efficacy, hope, optimism, and resilience. Items are rated using a 6-point Likert scale ranging from 1 (“strongly disagree) to 6 (“strongly agree”).

PES-NWI-12 - Practice Environment Scale of the Nursing Work Index (Lake 2002) [143]

30-item questionnaire measuring nurses' perception of the work environment. The tool is divided into five subscales consisting of: (1) nurse participation in hospital affairs (9 items); (2) nursing foundations for quality of care (10 items); (3) nurse manager ability, leadership, and support for nurses (5 items); (4) staffing and resource adequacy (4 items); and (5) collegial nurse-physician relations (3 items). Items are rated using a 4-point Likert scale ranging from 1 (‘strongly disagree’) to 4 (‘strongly agree’).

PFQ - Pandemic Fatigue Questionnaire (Labrague & Ballad 2021) [144]

10-item questionnaire measuring clinical nurses’ mental and physical fatigue associated with the COVID-19 pandemic. Items are rated using a 6-point Likert scale ranging from 0 (‘never’) to 5 (‘always’).

PHQ-9 - Patient Health Questionnaire (Kroenke & Spitzer 1999) [145]

9-item multipurpose instrument for screening, diagnosing, monitoring, and measuring the severity of depression. Items are rated using a 4-point Likert scale ranging from 0 (‘not at all’) to 4 (‘nearly every day’).

PHQ-4 - Patient Health Questionnaire (Kroenke et al. 2009) [146]

4-item multipurpose instrument for screening, diagnosing, monitoring, and measuring the severity of depression. Items are rated using a 4-point Likert scale ranging from 0 (‘not at all’) to 3 (‘nearly every day’).

PHPQ - Preparedness of Hospital Practice Questionnaire (Manaf et al. 2016) [147]

53-item questionnaire assessing preparedness in the areas of IT skills (7 items), interpersonal skills (7 items), basic skills (7 items), holistic skills (6 items), coping skills (4 items), ethic and legal skills (4 items), patient management skills (5 items), scientific knowledge (4 items), and clinical skills (3 items). Items are rated using a 5-point Likert scale ranging from 1 (‘very inadequate’) to 5 (‘very adequate’).

POS - Perceived Organizational Support (Eisenberger et al. 1987) [148]

8-item questionnaire measuring nurses’ opinions on the extent to which their workplace recognizes and values their well-being. Items are rated using a 5-point Likert scale ranging from 1 (‘strongly disagree’) to 5 (‘strongly agree’).

ProQOL-5 - Professional Quality of Life Scale Version 5 (Stamm 2010) [149]

30-item self-report questionnaire measuring compassion fatigue, work satisfaction, and burnout in professionals. Items are rated using a 5-point Likert scale ranging from 1 (‘never’) to 5 (‘very often’).

PSQI - Pittsburgh Sleep Quality Index (Buysse et al. 1989) [150]

19-item self-report assessing sleep quality and disturbances over a 1-month time interval. Items belong to one of seven subcategories: subjective sleep quality, sleep latency, sleep duration, habitual sleep efficiency, sleep disturbances, use of sleeping medication, and daytime dysfunction.

PSS-10 - Perceived Stress Scale (Cohen et al. 1983) [10]

10-item self-report questionnaire measuring perceived stress levels as experienced by an individual in daily life over the past month and eliciting stress symptoms. Items are rated using a 5-point Likert scale ranging from 0 (‘never’) to 4 (‘very often”).

PSSQ - Perceived Social Support Questionnaire (Lin et al. 2019) [151]

6-item scale measuring extent of nurse agreement with each item pertaining to perceived social support. Items are rated using a 5-point Likert scale ranging from 1 (‘strongly disagree’) to 5 (‘strongly agree’).

PWB - Psychological Wellbeing Scale (Ryff, 1989) [152]

42-item questionnaire measuring six aspects of wellbeing and happiness: autonomy, environmental mastery, personal growth, positive relations with others, purpose in life, and self-acceptance. Items are rated using a 7-point Likert scale ranging from 1 (‘strongly agree’) to 7 (‘strongly disagree’).

PTSS-10 - Post-Traumatic Symptom Scale-10 (Raphael et al. 1989) [153]

10‐item self‐report questionnaire assessing the presence and intensity of PTSD symptoms: sleeping difficulties, nightmares, feeling dejected or downtrodden, jumpiness or startled reactions, the need to withdraw from others, irritability and agitation, frequent mood swings, bad conscience, feelings of guilt, fear of places that remind an individual of the place that he/she works in, and muscular tension. Each of the 10 symptoms are rated using a 4‐point Likert scale ranging from 0 (‘not at all/never’) to 3 (‘very often’).

RAW-S - Resilience at Work (Winwood et al. 2013) [154]

24-item questionnaire within seven subscales used to measure resilience levels.

RBD - Risk Behavior Diagnostic Scale (Witte 1996) [155]

3-item questionnaire measuring perceived severity of COVID-19.

RBST - Rapid Burnout Screening Tool (Ong 2021) [156]

4-item questionnaire measuring burnout. Each dimension of burnout (emotional exhaustion, depersonalization, and reduced personal accomplishment) was assessed via one question. A fourth question ("how often do you feel burnt out from your work?”) provided a global assessment. Items were rated using a 5-point Likert scale.

RDAS - Revised Dyadic Adjustment Scale (Busby et al. 1995) [157]

14-item self-report questionnaire assessing seven dimensions of couple relationships within three overarching categories including consensus in decision making, values and affection, satisfaction in the relationship with respect to stability and conflict regulation, and cohesion as seen through activities and discussion. Items are rated using 6-point and 5-point Likert scales.

RS - Resilience Scale (Wagnild & Young 1987) [158]

25-item questionnaire measuring resilience. Items are scored using a 7-point Likert scale ranging from 1 (‘strongly disagree’) to 7 (‘strongly agree’).

SCL-90-R - Symptom Checklist-90-Revised (Derogatis 1992) [159]

90-item questionnaire assessing nine symptom dimensions: somatization, obsessive-compulsive, interpersonal sensitivity, depression, anxiety, hostility, phobic anxiety, paranoid ideation, and psychoticism. It is designed to provide an overview of a patient's symptoms and their intensity at a specific point in time. Items are rated using a 5-point Likert scale ranging from 0 (‘not at all’) to 4 (‘extremely’).

SCM-SF - Self Compassion Scale - Short Form (Neff 2011) [160]

12-item self-report questionnaire measuring self-compassion, defined as being open and kind to oneself and taking a non-judgmental attitude towards difficult feelings or thoughts. Items are rated using a 5-point Likert scale ranging from 1 (‘almost never’) to 5 (‘almost always’).

SF-36 - Short Form Survey (Ware 1992) [161]

36-item questionnaire measuring quality-of-life using eight scales: physical functioning (10 items), role limitations due to physical health (4 items), role limitations due to emotional problems (4 items), energy/fatigue, emotional well-being, social functioning (2 items), pain (2 items), and general health.

SQS - Sleep Quality Scale (Snyder et al. 2018) [162]

Single-item measuring quality of sleep in the past seven days. Items are rated using an 11-point Likert scale ranging from 0 (‘poor’) to 10 (‘excellent’).

SS - Stigma Scale (Park 2020) [163]

13-item questionnaire measuring perceived stigma of nurses regarding the MERS-CoV. Items are rated using a 5-point Likert scale.

SSS - Social Support Scale (House & Wells 1978) [164]

4-item scale measuring social support from the immediate supervisor, coworkers, spouse/partner, and friends/relatives. For these items, participants rate the extent to which each source fulfills a particular supportive function. Items are rated using a 4-point Likert scale ranging from 0 (‘not at all’) 3 (‘very much’).

STAI - State-Trait Anxiety Inventory (Spielberger 1970) [165]

40-item questionnaire consisting of 20 statements to evaluate state anxiety and 20 statements to evaluate trait anxiety. This anxiety test aims to measure two concepts of anxiety which are momentary/State anxiety (current state of anxiety) and basic/trait anxiety (stable aspects of anxiety including calmness, self-confidence, and a feeling of security). Items are rated using a 4-point Likert scale ranging from 1 (‘almost never’) to 4 (‘almost always’).

STAXI - State-Trait Anger Expression Inventory (Spielberger 1988) [166]

44-item questionnaire measuring anger both as an emotional situational response and a dispositional quality. It consists of six scales: trait anger, state anger, anger control, anger suppression, externally directed anger, and anger expression. Items are rated using a 4-point Likert scale ranging from 1 (‘I strongly disagree’) to 4 (‘I strongly agree’).

STSS - Secondary Traumatic Stress Scale (Bride et al. 2004) [167]

17-item questionnaire designed to measure the frequency of symptoms associated with secondary traumatic stress. It has three subscales: intrusion, arousal, and avoidance. Items are rated using a 5-point Likert scale ranging from 1 (‘never’) to 5 (‘very often’).

USMEQ-I - USM Emotional Quotient Inventory (Yusoff et al. 2010) [168]

13-item questionnaire measuring emotional intelligence. Items are rated using a 5-point Likert scale ranging from 0 (‘not like me’) to 4 (‘totally like me’).

WC - Ways of Coping (Folkman & Lazarus 1985) [169]

66-item questionnaire measuring how physicians cope with job-related stressors. Questionnaire is divided into eight component scales across two categories: problem-oriented coping strategies (confrontative coping behaviors, seeking social support, and planful problem solving) and emotion-oriented coping strategies (distancing, self-controlling, accepting responsibility, escape-avoidance behaviors, and positive reappraisal). Items are rated using a 4-point Likert-like scale ranging from 0 (‘not used’) to 3 (‘used a great deal’).

WSS - Workplace Stress Scale (The Marlin Company & the American Institute of Stress 2009) [170]

Eight statements describing how one feels in their job. The eight items are rated using a 5-point rating scale ranging from 1 (‘never’) to 5 (‘very often’).
