# Supplementary material for: Exploring Stress, Fatigue, Burnout, and Resilience Among Healthcare Personnel in Southern and South-Eastern Asia: A Scoping Review
Source: Public Health Rev. 2025 Nov 26;46:1608603. doi: 10.3389/phrs.2025.1608603 (PMC12690366; doi:10.3389/phrs.2025.1608603)
Supplement: Supplementary file 2 [file Table2.docx]

**Eligibility criteria**

To be included in the scoping review, studies were required to measure or focus on resilience, healthcare personnel, and Southeastern Asian countries. Peer-reviewed journal articles were eligible for inclusion if they were published in English between 2016 and 2022. Studies were excluded if full-text articles were unavailable or if their primary focus was on the resilience of health systems. Additionally, book chapters, conference papers, theses, unpublished clinical trials, and withdrawn papers were excluded from this review.

**Information Sources**

To identify potentially relevant documents, a comprehensive search of bibliographic databases was conducted from 2016 to December 2022. The databases included PubMed, PsycInfo, EMBASE, Scopus, Web of Science, CINAHL, and the Cochrane Library. An experienced librarian, Wendy Wu, drafted the search strategies and subsequently discussed search terms with a core team member. The finalized search strategy for PubMed is provided in the Additional Files. The search results were exported into EndNote and subsequently imported into Covidence, a systematic review management and screening software. Covidence automatically removed duplicate records. In addition to the electronic database search, supplementary searches were performed in the grey literature (e.g., Google and the World Health Organization website), and relevant studies were identified through manual screening of reference lists from pertinent reviews and articles.

**Selection of sources of evidence**

To enhance consistency among reviewers, four reviewers, working in pairs, screened 8,918 publications based on titles and abstracts. Subsequently, 404 full-text articles were evaluated and screened by defined inclusion and exclusion criteria. 55 articles were selected for data extraction. Discrepancies in study selection and data extraction were resolved through consensus and discussion among the reviewers.

| Searches | Search Terms | Results |
| --- | --- | --- |
| Search 1 | Health Personnel[Mesh:NoExp] OR Allied Health Personnel[Mesh] OR Anesthetists[Mesh] OR Caregivers [MeSH] OR Case Managers [MeSH] OR Coroners and Medical Examiners [MeSH] OR Dental Staff [MeSH] OR Dentists [MeSH] OR Doulas [MeSH] OR Epidemiologists[MeSH] OR Faculty, Dental[MeSH] OR Faculty, Medical [MeSH] OR Faculty, Nursing [MeSH] OR Health Facility Administrators [MeSH] OR Infection Control Practitioners [MeSH] OR Medical Chaperones [MeSH] OR Laboratory Personnel [MeSH] OR Medical Staff [MeSH] OR Nurses [MeSH] OR Nursing Staff [MeSH] OR Nutritionists [MeSH] OR Occupational Therapists [MeSH] OR Optometrists [MeSH] OR Personnel, Hospital [MeSH] OR Pharmacists [MeSH] OR Physical Therapists [MeSH] OR Physician Executives [MeSH] OR Physicians [MeSH] OR Health Personnel [Title/Abstract] OR Health Worker* [Title/Abstract] OR Dental Auxiliar* [Title/Abstract] OR Dental Assistant* [Title/Abstract] OR Dental Hygienist* [Title/Abstract] OR Dental Technician*[Title/Abstract] OR Denturist* [Title/Abstract] OR Emergency Medical Technician* [Title/Abstract] OR Home Health Aid* [Title/Abstract] OR Medical Record Administrator* [Title/Abstract] OR Medical Secretar*[Title/Abstract] OR Medical Receptionist*[Title/Abstract] OR Nursing Assistant*[Title/Abstract] OR Psychiatric Aid*[Title/Abstract] OR Operating Room Technician*[Title/Abstract] OR Pharmacy Technician*[Title/Abstract] OR Physical Therapist Assistant*[Title/Abstract] OR Physician Assistant*[Title/Abstract] OR Ophthalmic Assistant*[Title/Abstract] OR Pediatric Assistant*[Title/Abstract] OR Doctor Assistant*[Title/Abstract] OR Doctors Assistant*[Title/Abstract] OR Anesthetist*[Title/Abstract] OR Anesthesiologist*[Title/Abstract] OR Caregiver* [Title/Abstract] OR Case Manager*[Title/Abstract] OR Medical Examiner* [Title/Abstract] OR Dentist* [Title/Abstract] OR Endodontist* [Title/Abstract] OR Orthodontist* [Title/Abstract] OR Doula* [Title/Abstract] OR Epidemiologist* [Title/Abstract] OR dental Faculty [Title/Abstract] OR medical Faculty [Title/Abstract] OR nursing Faculty [Title/Abstract] OR Health Facility Administrator* [Title/Abstract] OR Health Facility workers* [Title/Abstract] OR Health Facility personnel [Title/Abstract] OR Health Facility staff [Title/Abstract] OR Hospital Administrator* [Title/Abstract] OR Hospital worker* [Title/Abstract] OR Hospital personnel [Title/Abstract] OR Medical Staff [Title/Abstract] OR Medical worker* [Title/Abstract] OR Medical personnel [Title/Abstract] OR medical expert* [Title/Abstract] OR hospital Chief Executive Officer* [Title/Abstract] OR hospital Executive Officer* [Title/Abstract] OR hospital Officer* [Title/Abstract] OR Medical Chaperone* [Title/Abstract] OR Laboratory Personnel [Title/Abstract] OR Medical Laboratory worker* [Title/Abstract] OR Medical Laboratory staff [Title/Abstract] OR Medical Laboratory administrator* [Title/Abstract] OR hospital Staff [Title/Abstract] OR Hospitalist* [Title/Abstract] OR Nurses [Title/Abstract] OR nurse [Title/Abstract] OR Nurse Administrator* [Title/Abstract] OR Nurse Practitioner* [Title/Abstract] OR Nurse Specialist* [Title/Abstract] OR Nurse Clinician* [Title/Abstract] OR Nurse Midwife [Title/Abstract] OR Nursing Staff [Title/Abstract] OR Nursing personnel [Title/Abstract] OR Nutritionist* [Title/Abstract] OR Therapist* [Title/Abstract] OR Optometrist* [Title/Abstract] OR Dental Staff [Title/Abstract] OR Hospital Volunteer* [Title/Abstract] OR Pharmacist* [Title/Abstract] OR Physician Executive* [Title/Abstract] OR Physician* [Title/Abstract] OR Allergist* [Title/Abstract] OR Cardiologist* [Title/Abstract] OR Dermatologist* [Title/Abstract] OR Endocrinologist* [Title/Abstract] OR Medical Graduates [Title/Abstract] OR Gastroenterologist* [Title/Abstract] OR Practitioner* [Title/Abstract] OR Geriatrician* [Title/Abstract] OR healthcare personnel [Title/Abstract] OR health care personnel [Title/Abstract] OR resident* [Title/Abstract] OR fellow* [Title/Abstract] OR acupuncturist* [Title/Abstract] OR advanced practice provider* [Title/Abstract] OR anesthesist* [Title/Abstract] OR care coordinator* [Title/Abstract] OR dental personnel [Title/Abstract] OR periodontist* [Title/Abstract] OR prosthodontist* [Title/Abstract] OR emergency medical dispatcher* [Title/Abstract] OR eye care professional* [Title/Abstract] OR ophthalmologist* [Title/Abstract] OR optician* [Title/Abstract] OR orthoptist*[Title/Abstract] OR health auxiliar* [Title/Abstract] OR healthcare assistant* [Title/Abstract] OR health care assistant* [Title/Abstract] OR health educator* [Title/Abstract] OR childbirth educator* [Title/Abstract] OR diabetes educator* [Title/Abstract] OR health workforce [Title/Abstract] OR medical assistant* [Title/Abstract] OR medical registrar* [Title/Abstract] OR general practice registrar* [Title/Abstract] OR specialist registrar* [Title/Abstract] OR surgical registrar* [Title/Abstract] OR medical specialist* [Title/Abstract] OR military medical personnel [Title/Abstract] OR andrologist* [Title/Abstract] OR Electrophysiologist* [Title/Abstract] OR dermatovenereologist* [Title/Abstract] OR diabetologist* [Title/Abstract] OR epileptologist* [Title/Abstract] OR fertility specialist* [Title/Abstract] OR gerontologist* [Title/Abstract] OR gynecologist* [Title/Abstract] OR oncologist* [Title/Abstract] OR Hematologist* [Title/Abstract] OR hepatologist* [Title/Abstract] OR immunologist* [Title/Abstract] OR infectious disease specialist* [Title/Abstract] OR intensivist* [Title/Abstract] OR internist* [Title/Abstract] OR medical geneticist* [Title/Abstract] OR neonatologist* [Title/Abstract] OR Nephrologist* [Title/Abstract] OR Neurologist* [Title/Abstract] OR obstetrician* [Title/Abstract] OR orthopedic specialist* [Title/Abstract] OR Otolaryngologist* [Title/Abstract] OR Pathologist* [Title/Abstract] OR Pediatrician* [Title/Abstract] OR phlebologist* [Title/Abstract] OR physiatrist* [Title/Abstract] OR podiatrist* [Title/Abstract] OR psychiatrist* [Title/Abstract] OR paediatric specialist* [Title/Abstract] OR Pulmonologist* [Title/Abstract] OR Radiologist* [Title/Abstract] OR Rheumatologist* [Title/Abstract] OR Neurosurgeon* [Title/Abstract] OR Urologist* [Title/Abstract] OR Urogynecologist* [Title/Abstract] OR vaccinologist* [Title/Abstract] OR psychotherapist* [Title/Abstract] OR medical scribe [Title/Abstract] OR nursing home personnel [Title/Abstract] OR nursing home worker* [Title/Abstract] OR nursing home staff [Title/Abstract] OR nursing home assistant* [Title/Abstract] OR nursing home administrator* [Title/Abstract] OR paramedical personnel [Title/Abstract] OR perfusionist* [Title/Abstract] OR personal trainer* [Title/Abstract] OR dietician* [Title/Abstract] OR prosthetist* [Title/Abstract] OR traditional healer* [Title/Abstract] OR transplant coordinator* [Title/Abstract] OR health care provider* [Title/Abstract] OR healthcare provider* [Title/Abstract] OR Healthcare staff [Title/Abstract] OR Health care staff [Title/Abstract] OR healthcare worker* [Title/Abstract] OR health care worker* [Title/Abstract] OR Health Care Professional* [Title/Abstract] OR Healthcare Professional* [Title/Abstract] OR medical service personnel [Title/Abstract] OR technician* [Title/Abstract] OR phlebotomist* [Title/Abstract] OR Pathologists' Assistant* [Title/Abstract] OR trainee* [Title/Abstract] OR Laboratory Scientist* [Title/Abstract] OR Laboratory Technician* [Title/Abstract] OR Laboratory Assistant* [Title/Abstract] OR clinical workforce [Title/Abstract] OR autopsy personnel [Title/Abstract] OR clerical personnel [Title/Abstract] OR clerical staff [Title/Abstract] OR clerical worker* [Title/Abstract] OR dietary personnel [Title/Abstract] OR dietary worker* [Title/Abstract] OR dietary staff [Title/Abstract] OR clerical worker* [Title/Abstract] OR clerical staff [Title/Abstract] OR clerical personnel [Title/Abstract] OR environmental services personnel [Title/Abstract] OR environmental services staff [Title/Abstract] OR environmental services worker* [Title/Abstract] OR laundry personnel [Title/Abstract] OR laundry worker* [Title/Abstract] OR laundry staff [Title/Abstract] OR security staff [Title/Abstract] OR security personnel [Title/Abstract] OR security worker* [Title/Abstract] OR maintenance staff [Title/Abstract] OR maintenance worker* [Title/Abstract] OR maintenance personnel [Title/Abstract] OR facilities management personnel [Title/Abstract] OR facilities management staff [Title/Abstract] OR facilities management worker* [Title/Abstract] OR administrative staff [Title/Abstract] OR administrative personnel [Title/Abstract] OR administrative worker* [Title/Abstract] OR billing personnel [Title/Abstract] OR billing staff [Title/Abstract] OR volunteer personnel [Title/Abstract] OR volunteer worker* [Title/Abstract] OR Psychiatric Hospital Staff [Title/Abstract] OR Psychologist* [Title/Abstract] OR Social Worker* [Title/Abstract] OR Psychiatrist* [Title/Abstract] OR Psychotherapist* [Title/Abstract] | 609,590 |
| Search 2 | "Workplace/psychology" [MeSH] OR "Resilience, Psychological" [MeSH] OR "Attitude of Health Personnel" [MeSH] OR "Adaptation, Psychological" [MeSH] OR Stress, Psychological [MeSH] OR Occupational Stress/psychology[MeSH] OR Burnout, Professional [MeSH] OR depression [mesh] OR fatigue[mesh] OR psychological adaptation [Title/Abstract] OR coping[Title/Abstract] OR resilience [Title/Abstract] OR resiliency [Title/Abstract] OR resilient [Title/Abstract] OR hardness [Title/Abstract] OR resistance [Title/Abstract] OR stress [Title/Abstract] OR moral injury [Title/Abstract] OR psychological Distress [Title/Abstract] OR depression [Title/Abstract] OR anxiety [Title/Abstract] OR psychologic impact* [Title/Abstract] OR burnout [Title/Abstract] OR Exhaustion [Title/Abstract] OR disillusionment [Title/Abstract] OR anger [Title/Abstract] OR frustration [Title/Abstract] OR grief [Title/Abstract] OR Adaptive Behavior* [Title/Abstract] OR Emotional Adjustment [Title/Abstract] OR [Psychological Endurance](javascript:XslPostBack('ctl00$ctl00$MainContentArea$MainContentArea$xslResults','ThesaurusLink','LinkTarget%7CauthorityDetail%24LinkTerm%7CDE%2B%2522Psychological%2BEndurance%2522');) [Title/Abstract] OR Strengths-Based Intervention* [Title/Abstract] OR Emotional Stability [Title/Abstract] OR optimism [Title/Abstract] OR Psychological Capital [Title/Abstract] OR fatigue [Title/Abstract] OR Caregiver Burden[Title/Abstract] OR Tiredness [Title/Abstract] | 908,705 |
| Search 3 | “Asia, Southeastern"[Mesh] OR Borneo [Title/Abstract] OR Brunei [Title/Abstract] OR Cambodia [Title/Abstract] OR Indochina [Title/Abstract] OR Indonesia [Title/Abstract] OR Laos [Title/Abstract] OR Malaysia [Title/Abstract] OR Mekong Valley [Title/Abstract] OR Myanmar [Title/Abstract] OR Philippines [Title/Abstract] OR Singapore [Title/Abstract] OR Thailand [Title/Abstract] OR Timor-Leste [Title/Abstract] OR Vietnam [Title/Abstract] OR "Asia, Western"[Mesh:NoExp] OR Bangladesh [MeSH] OR Bhutan [MeSH] OR Bangladesh [Title/Abstract] OR Bhutan [Title/Abstract] OR India [MeSH] OR India [Title/Abstract] OR Afghanistan [MeSH] OR Afghanistan [Title/Abstract] OR Nepal [MeSH] OR Pakistan [MeSH] OR Sri Lanka [MeSH] OR Nepal [Title/Abstract] OR Pakistan [Title/Abstract] OR Sri Lanka [Title/Abstract] OR "Democratic People's Republic of Korea"[Mesh] OR “Democratic People's Republic of Korea"[Title/Abstract] OR North Korea [Title/Abstract] OR Maldives [MeSH] OR Maldives [Title/Abstract] OR "southeastern asia"[Title/Abstract] OR "south eastern asia"[Title/Abstract] OR "southeast asia"[Title/Abstract] OR "south east asia"[Title/Abstract] | 538,208 |
| Search 5 | #1 AND #2 AND #3 | 6,465 |
| Search 6 | comment [Publication Type] OR editorial [Publication Type] OR letter[Publication Type] OR News[Publication Type] | 600,594 |
| Search 7 | #5 NOT #6 | 6,303 |
| Search 8 | Limit to English | 6,291 |
