# Supplementary material for: Exploring Stress, Fatigue, Burnout, and Resilience Among Healthcare Personnel in Southern and South-Eastern Asia: A Scoping Review
Source: Public Health Rev. 2025 Nov 26;46:1608603. doi: 10.3389/phrs.2025.1608603 (PMC12690366; doi:10.3389/phrs.2025.1608603)
Supplement: Supplementary file 3 [file Table3.docx]

**Burnout** - physical, emotional, or mental exhaustion accompanied by decreased motivation, lowered performance, and negative attitudes toward oneself and others. It results from performing at a high level until stress and tension, especially from extreme and prolonged physical or mental exertion or an overburdening workload, take their toll [6]

**Fatigue** - a state of tiredness and diminished functioning. Fatigue is typically a normal, transient response to exertion, stress, boredom, or inadequate sleep but also may be unusually prolonged and indicative of disorder (e.g., chronic fatigue syndrome, anemia, hypothyroidism) [100]

**Health** - Health is a state of complete physical, mental and social well-being and not merely the absence of disease or infirmity [1]

**Healthcare personnel (HCP)** - all paid and unpaid persons serving in healthcare settings who have the potential for direct or indirect exposure to patients or infectious materials, including body substances (e.g., blood, tissue, and specific body fluids); contaminated medical supplies, devices, and equipment; contaminated environmental surfaces; or contaminated air. These HCP may include, but are not limited to, emergency medical service personnel, nurses, nursing assistants, physicians, technicians, therapists, phlebotomists, pharmacists, students and trainees, contractual staff not employed by the health care facility, and persons (e.g., clerical, dietary, environmental services, laundry, security, maintenance, engineering and facilities management, administrative, billing, and volunteer personnel) not directly involved in patient care but potentially exposed to infectious agents that can be transmitted among from HCP and patients. For this update, HCP does not include dental healthcare personnel, autopsy personnel, and laboratory personnel, as recommendations to address occupational infection prevention and control (IPC) services for these personnel are posted elsewhere [18]

**Mindfulness** - awareness of one’s internal states and surroundings. The concept has been applied to various therapeutic interventions—for example, mindfulness-based cognitive behavior therapy, mindfulness-based stress reduction, and mindfulness meditation—to help people avoid destructive or automatic habits and responses by learning to observe their thoughts, emotions, and other present-moment experiences without judging or reacting to them [82]

**Resilience** - the process and outcome of successfully adapting to difficult or challenging life experiences, especially through mental, emotional, and behavioral flexibility and adjustment to external and internal demands. A number of factors contribute to how well people adapt to adversities, predominant among them (a) the ways in which individuals view and engage with the world, (b) the availability and quality of social resources, and (c) specific coping strategies [2]

**South-eastern Asian and Southern Asian (United Nations)** – The United Nations (UN) includes these 20 countries as belonging to Southeast Asia: Brunei, Cambodia, Indonesia, Laos, Malaysia, Myanmar, Philippines, Singapore, Thailand, Timor-Leste, Vietnam, Afghanistan, Bangladesh, Bhutan, India, Iran, Maldives, Nepal, Pakistan, and Sri Lanka [20]

**South-eastern Asian and Southern Asian (World Health Organization)**

The World Health Organization (WHO) includes these 11 countries as belonging to Southeast Asia: Bangladesh, Bhutan, Democratic People’s Republic of Korea, India, Indonesia, Maldives, Myanmar, Nepal, Sri Lanka, Thailand, and Timor-Leste [21].

**Stress** - the physiological or psychological response to internal or external stressors. Stress involves changes affecting nearly every system of the body, influencing how people feel and behave [101]
